# Convergent Synthesis of Template‐Assembled Synthetic G‐Quartets (TASQs) Used as Biomimetic and Multivalent G‐Quadruplex (G4) Ligands

**DOI:** 10.1002/chem.202503002

**Published:** 2025-11-20

**Authors:** Sandy Raevens, Cécile Desingle, Marc Pirrotta, Francesco Rota Sperti, Andréa Pieri, Pauline Lejault, Ibai E. Valverde, David Monchaud

**Affiliations:** ^1^ ICMUB CNRS UMR6302 Université Bourgogne Europe (UBE) Dijon 21078 France

**Keywords:** G‐quadruplexes, Synthesis design, macrocycles, chemical biology, molecular tools, synthetic G‐quartet, TASQ

## Abstract

Precise insights into the biology of DNA and RNA G‐quadruplexes (G4s) were obtained thanks to the use of ever more versatile G4‐interacting molecules (or G4 ligands), which bear additional moieties making them functionalizable in situ (i.e., while in interaction with cellular G4s) thanks to bioorthogonal chemistry. We recently reported on a series of biomimetic G4 ligands known as TASQs (for template‐assembled synthetic G‐quartets) whose multifunctionality was exploited to visualize (pre‐targeted and in situ click imaging) and identify cellular G4s (G4omics and Chem‐CLIP). We report herein on a novel synthetic strategy that makes multivalent TASQs readily accessible and opens brand new perspectives for the design of ever smarter molecular tools to unravel the quite complex G4 biology in a more accurate, straightforward, and simpler manner.

## Graphical abstract

The family of template‐assembled synthetic G‐quartets, or TASQs, comprises different molecular tools that have been successfully used to decipher G‐quadruplex (G4) biology (*via* optical imaging and/or affinity precipitation). The synthetic access to the different TASQs has been thoroughly revisited herein to make it simpler and more efficient thanks to a convergent strategy that relies on an unprecedented key common intermediate.

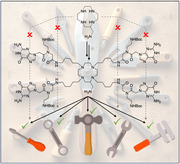

## 1 Introduction

Template‐assembled synthetic G‐quartets (or TASQs) were first reported by Nikan & Sherman in 2008 [1]. These structurally complex molecules were built on the principle of Template‐Assembled Synthetic Proteins (TASPs) devised by Mutter & Vuilleumier as a way to control polypeptide folding into functional protein‐like macrobiomolecules [2, 3]. TASQs were developed to assemble discrete G‐quartets intramolecularly: to this end, four guanine (G) residues were covalently linked to a template (a Cram's cavitand) [4] by click chemistry. The resulting molecules were found to be conformationally dynamic, adopting a so‐called ‘open’ conformation in which the Gs are independent of each other and a ‘closed’ conformation in which the synthetic G‐quartet is folded *via* the formation of eight hydrogen bonds (involving both Watson‐Crick and Hoogsteen faces) [5, 6]. This first generation of TASQs was built to study the chelation of cations (Na^+^, K^+^, Sr^2+^) on the open ⇆ closed conformation equilibrium in organic media by nuclear magnetic resonance (NMR) [1, 7].

Soon after, strategies were devised to obtain water‐soluble TASQs by employing hydrophilic templates: Stefan *et al.* used a 1,4,7,10‐tetraazacyclododecane‐*N,N‘,N’‘,N’‘’*‐tetraacetic acid (DOTA) template [8], while Murat *et al.* used a regioselectively addressable functionalized template (RAFT, initially introduced by Mutter for the synthesis of TASP) [9], and Bare *et al.* used a cavitand with phosphate appendages [10]. These efforts were invested to achieve different goals: the DOTA‐based TASQs were intended to be used as biomimetic G‐quadruplex ligands (or G4 ligands, *vide infra*) while the RAFT‐based TASQs to further study their conformational dynamism, and the cavitand‐based TASQs to evaluate the binding affinity of G‐quartet‐interacting molecules in vitro.

**FIGURE 1:**
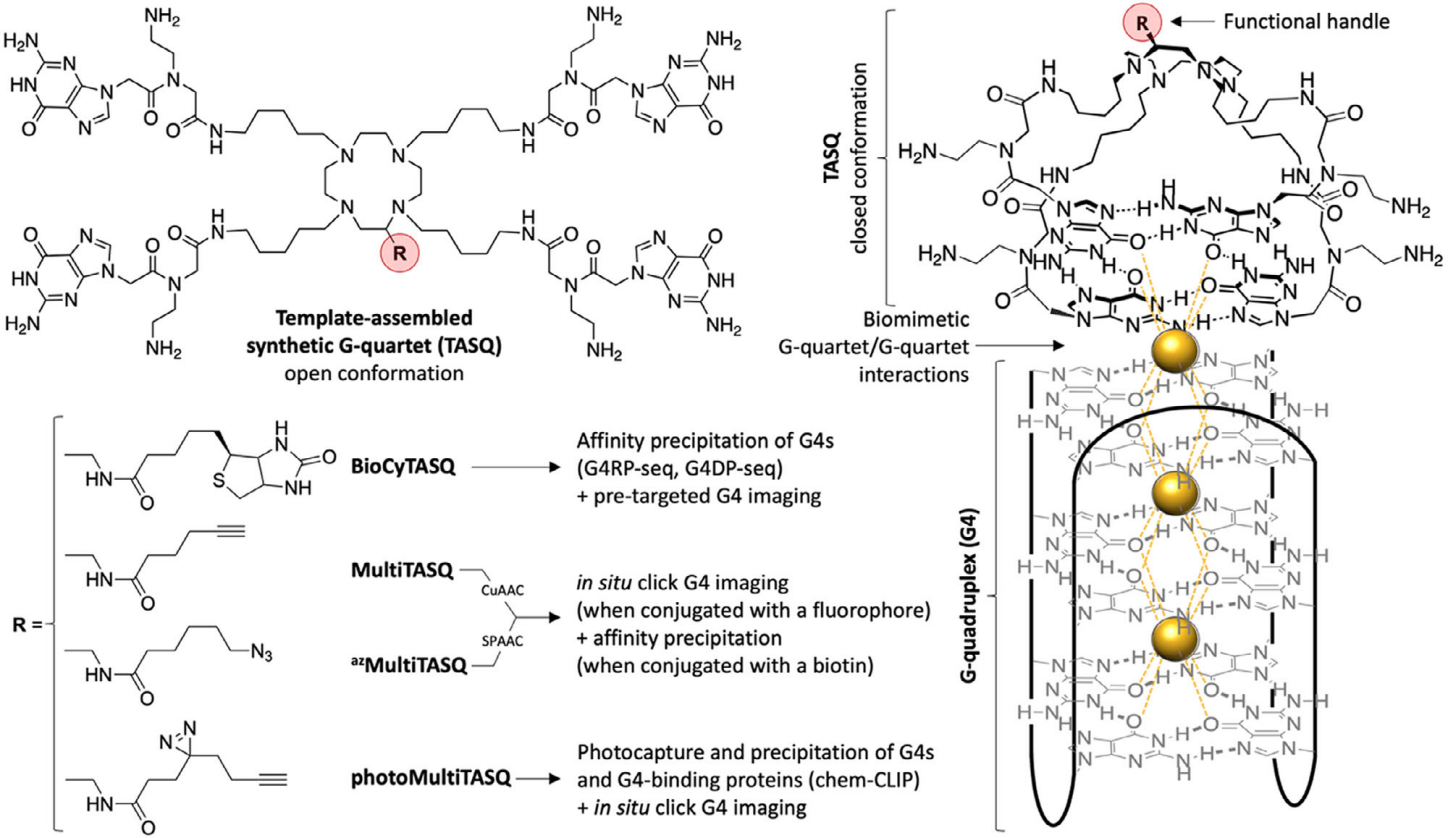
General chemical structure of the TASQs (along with their applications as a function of the nature of the functional handle **R**), represented under their open (upper left panel) and closed conformation (right panel) upon interaction with a G‐quadruplex (G4), driven by the nature‐inspired self‐interaction of G‐quartets (one from the TASQ, one from the G4). Golden spheres represent physiological cations (K^+^, Na^+^).

The application that has undoubtedly been studied the most is the use of TASQs as biomimetic G4‐ligands [11, 12]. To date, a dozen different prototypes of TASQs have been developed and used in different applications (Figure 1): from biomimetic (**DOTASQ** and **PorphySQ**) [8, 13] to smart G4 ligands (**^PNA^DOTASQ**, **^PNA^PorphySQ,** and **TriazoTASQ**) [14, 15, 16], from twice‐as‐smart fluorescent G4 probes (**PyroTASQ**, **N‐TASQ,** and **^Tz^NTASQ**) [17, 18, 19] to multifunctional ligands, being either biotinylated (**BioTASQ** and **BioCyTASQ**) [20, 21, 22], clickable (**MultiTASQ** and **^az^MultiTASQ**) [23], or photoactivatable TASQs (**photoMultiTASQ**) [24]. The latter family of ligands, the so‐called multifunctional ligands, were specially tailored to investigate G4 cellular biology. Biotinylated TASQs have proved to be very useful molecular baits to fish G4s out of cell lysates in protocols named G4RP‐seq for RNA G4s (for G4‐RNA precipitation and sequencing) [22, 25], and G4DP‐seq for DNA G4s (G4‐DNA precipitation and sequencing) [26]. Clickable TASQs can be used for both in situ click imaging and chemical cross‐linking and isolation by pull‐down (Chem‐CLIP) investigations (for reviews on these techniques, see [27, 28]).

The scope of applications that have been developed using water‐soluble TASQs explains why some of them have been made commercially available (**BioCyTASQ** and **MultiTASQ**). From a chemical point of view, the most versatile TASQs are being based on a *C*‐functionalized cyclen (*C*‐aminomethyl‐cyclen, or **AMC**) [29, 30], whose exocyclic primary amine allows for the introduction of a handle of interest, that is, a biotin for **BioTASQ** and **BioCyTASQ**, 5‐hexynoic acid for **MultiTASQ**, 6‐azido‐hexanoic acid for **^az^MultiTASQ,** and 3‐(3‐(but‐3‐yn‐1‐yl)‐3H‐diazirin‐3‐yl)propanoic acid for **photoMultiTASQ**. As further discussed below, the synthesis of these TASQs is quite tedious for two reasons: first, because most of the synthetic steps involve a quadruple functionalization, corresponding to the four arms of the TASQ; second, because the strategies implemented were linear, the first step being the modification of the **AMC** with the desired handle (which has, as we will see later, negative practical consequences). We reasoned that a convergent synthesis of TASQ could be devised to make TASQs' access far more strategically relevant, less time‐demanding, and less expensive. We report herein on this straightforward strategy, which allows for synthesizing water‐soluble and multivalent TASQs in a shorter and more efficient manner.

## 2 Results and Discussion

### 2.1 The Former Syntheses of TASQs and Proposed Improvements

**SCHEME 1:**
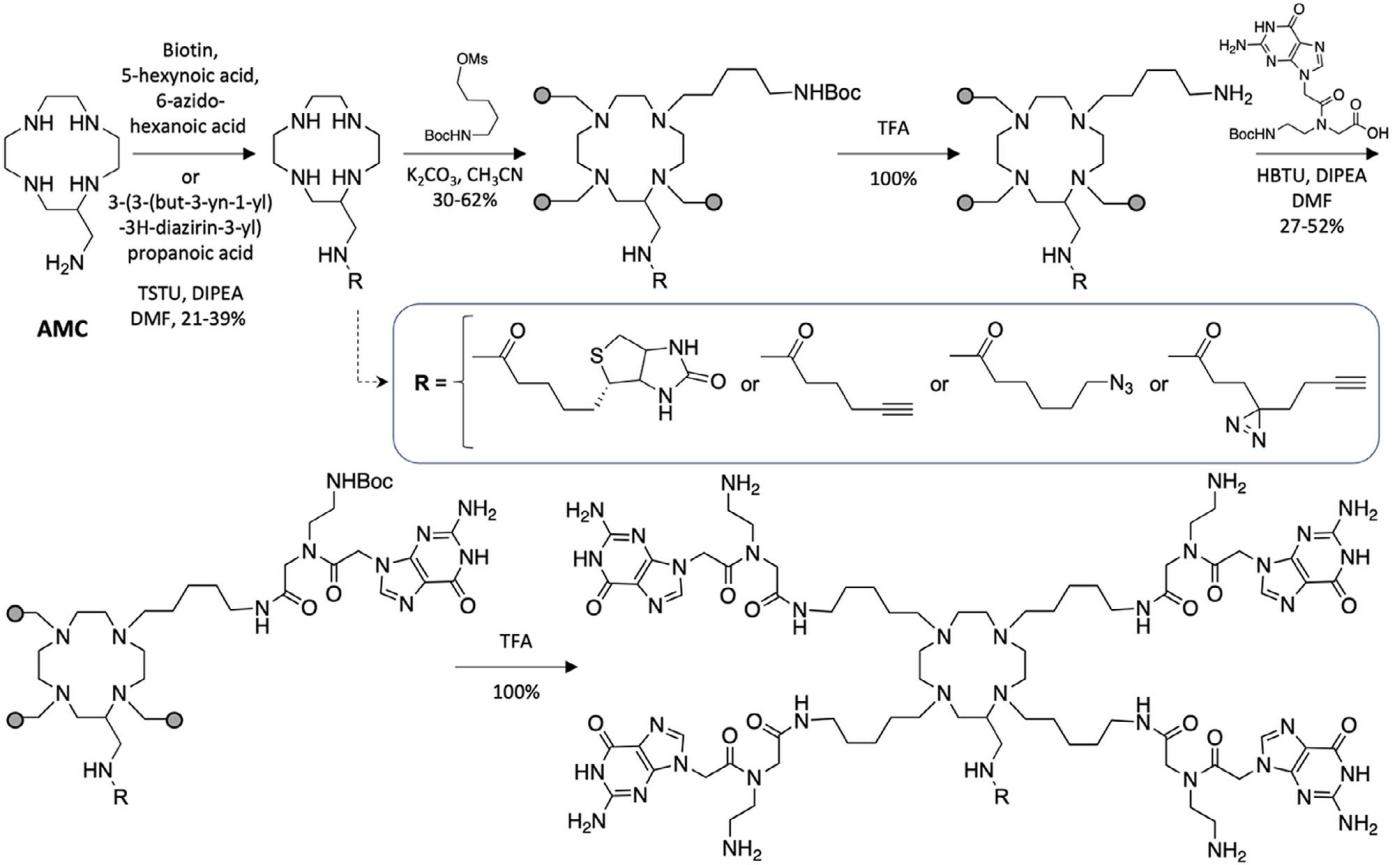
Former, linear syntheses of **BioCyTASQ**, **MultiTASQ**, **^az^MultiTASQ,** and **photoMultiTASQ** (overall chemical yields: 12.5, 6.2, 2.7, and 2.5% over 5 steps, respectively).

As indicated above, the synthesis of the multivalent TASQs starts with a coupling between **AMC** and the handles of interest (Scheme 1). While this strategy could be acceptable with biotin, 5‐hexynoic acid, and 6‐azido‐hexanoic acid, it turned out to be a critical issue with 3‐(3‐(but‐3‐yn‐1‐yl)‐3H‐diazirin‐3‐yl)propanoic acid for two reasons: first, its elevated cost (*ca*. 3 k€/g vs. < 0.2 k€/g for the other handles), which has to be associated with the low overall yield of the synthesis, making its introduction as the very first step irrelevant; second, its light sensitivity, which imposes a full synthesis in the dark.

A possible way to circumvent this pitfall would be to introduce the handle of interest at the end of the TASQ synthesis. To make this possible, the first step of the synthesis would be a protection of the primary amine of **AMC**. However, the synthetic access of TASQs involves both acidic and basic treatments, and the presence of a cyclen core precludes the use of metal‐based deprotection methods (such as catalytic hydrogenation). The reversible protection of **AMC** or *C*‐functionalized polyazamacrocycles has been sparsely described in the literature; given the necessity of finding a protecting group for primary amines that resists both acidic and basic conditions, our options were even more limited. An article from Amendola *et al.* reported on the protection of *C*‐aminomethyl‐13aneN4 using 1,8‐naphthalic anhydride (1.0 mol. equiv. of naphthalic anhydride in ethanol stirred at 85°C for 3 h) [31]. Although not described in the aforementioned article, the resulting naphthalimide could be easily removed using hydrazine hydrate [32, 33]. The chemical stability of the naphthalimide protecting group could make it suitable for our purpose; we thus opted for this strategy to protect **AMC**.

### 2.2 An Optimized Synthesis of AMC

**SCHEME 2:**
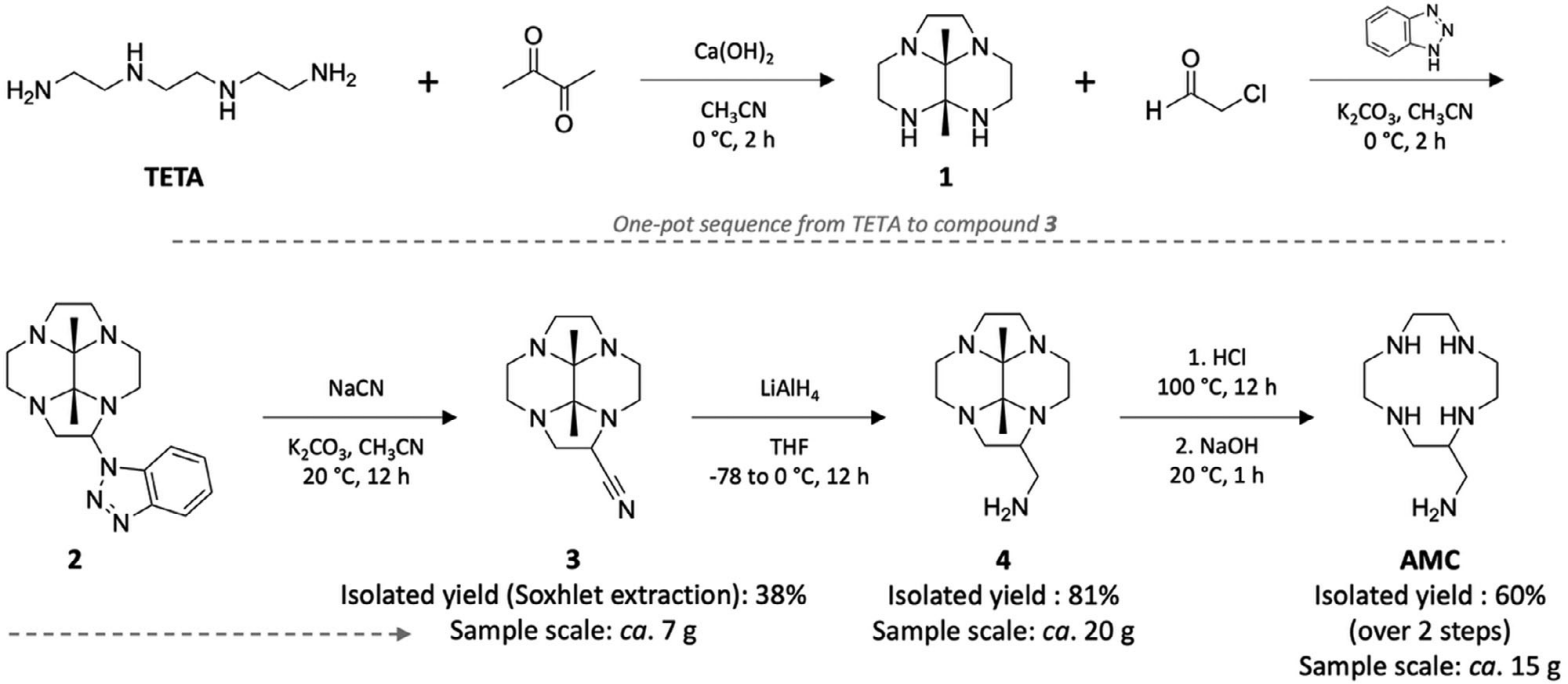
Optimized synthesis of **AMC** (overall chemical yield: 18.4% over 6 steps).

With the aim of optimizing the whole synthesis of multivalent TASQs, we first invested efforts to optimize the synthesis of the starting compound **AMC**. Its traditional synthesis (Scheme 2) starts with freshly recrystallized triethylenetetramine (or **TETA**), which was condensed with 2,3‐butanedione to afford a tricyclic **1**. Chloroacetaldehyde and benzotriazole (according to Katritzki's strategy) [34] were added to form the cyclen scaffold **2**; the benzotriazole moiety was then displaced using sodium cyanide to provide **3**, which was then purified (as further discussed below, this step is critical) prior to its reduction with LiAlH_4_, to provide **4**. The bis‐aminal bridge was then cleaved under strong acidic conditions to afford **AMC** as a hydrochloride salt. The free amine form is obtained after addition of NaOH and extraction with chloroform, which is usually performed prior to its use. This synthesis could be considered as poorly efficient since only 15 g of **AMC** are usually obtained from 140 g of **TETA** (12.6% overall chemical yield).

The main issue of this synthesis is the tedious purification of **3**, performed by column chromatography on alumina gel. Each fraction of this time‐consuming purification has to be carefully monitored (by NMR) to obtain **3** in the highest purity possible, which is required to guarantee the precipitation of the **AMC** hydrochloride salt at the end of this reaction sequence. To circumvent this issue, we found that n‐pentane efficiently solubilizes **3** only; we thus exploited this property by implementing a continuous solvent extraction technique using a Soxhlet apparatus. This extraction, carried out with the crude material adsorbed on alumina, led to the isolation of **3** with a high degree of purity, an improved chemical yield (from 26% to 38%), and better reproducibility. It also allowed lowering both the ecological cost (thanks to a *ca*. 20‐fold decrease in volume of solvents used; 650 mL vs. 15 L on a 140 g scale) and the analytical cost (a single NMR analysis is required at the end of the purification).

Overall, the global yield from **TETA** is still low (from 12.6 to 18.4%, despite a 1.5‐fold improvement), but the low cost of the starting materials, the reduced volumes required for the purification of **3**, the decreased number of analyses, and the increased reproducibility of the process make **AMC** access more reliable.

### 2.3 The Synthesis of the Common Intermediate TASQ‐NH_2_

We then attempted to protect the amine side chain of **AMC** according to the methodology described above [31]. Naphthalic anhydride successfully reacted with the exocyclic amine of **AMC** in EtOH from 0°C to room temperature (RT) overnight to provide the protected **AMC 5** in 57% chemical yield after purification by semi‐preparative HPLC. It is worth noting that, in our hands, the conditions described by Amendola *et al.* [31], that is, 85°C in EtOH for 3 h, led to the formation of by‐products by acylation of the intracyclic amines. Regioselective acylation of the primary amine was achieved only by decreasing the reaction temperature to 0°C. We then checked the reversibility of this protection step, treating **5** with hydrazine hydrate for 16 h in EtOH at reflux, described to be adapted to the naphthalimide protecting group [32, 33]: quite satisfyingly, the **AMC** was recovered in 52% chemical yield (unoptimized), which thus confirmed the suitability of this strategy to transiently protect the **AMC** template. It is worth noting that first attempts of regioselective protection of the exocyclic amine of **AMC** using phthalimide as a protective group were largely unsuccessful. Following a procedure reported in a patent [35], **AMC** was reacted with phthalic anhydride (slow addition (30 min) of 1.0 mol. equiv. of phthalic anhydride in cold isopropanol before refluxing the mixture at 83°C) for 16 h to afford mono‐, di‐, and tri‐acylated derivatives of **AMC**; however, the formation of the expected phthalimide was never observed by LCMS. In sharp contrast, the naphthalimide spontaneously forms after the reaction of the anhydride with the primary amine in polar protic solvents (EtOH, iPrOH), even at RT.

**SCHEME 3:**
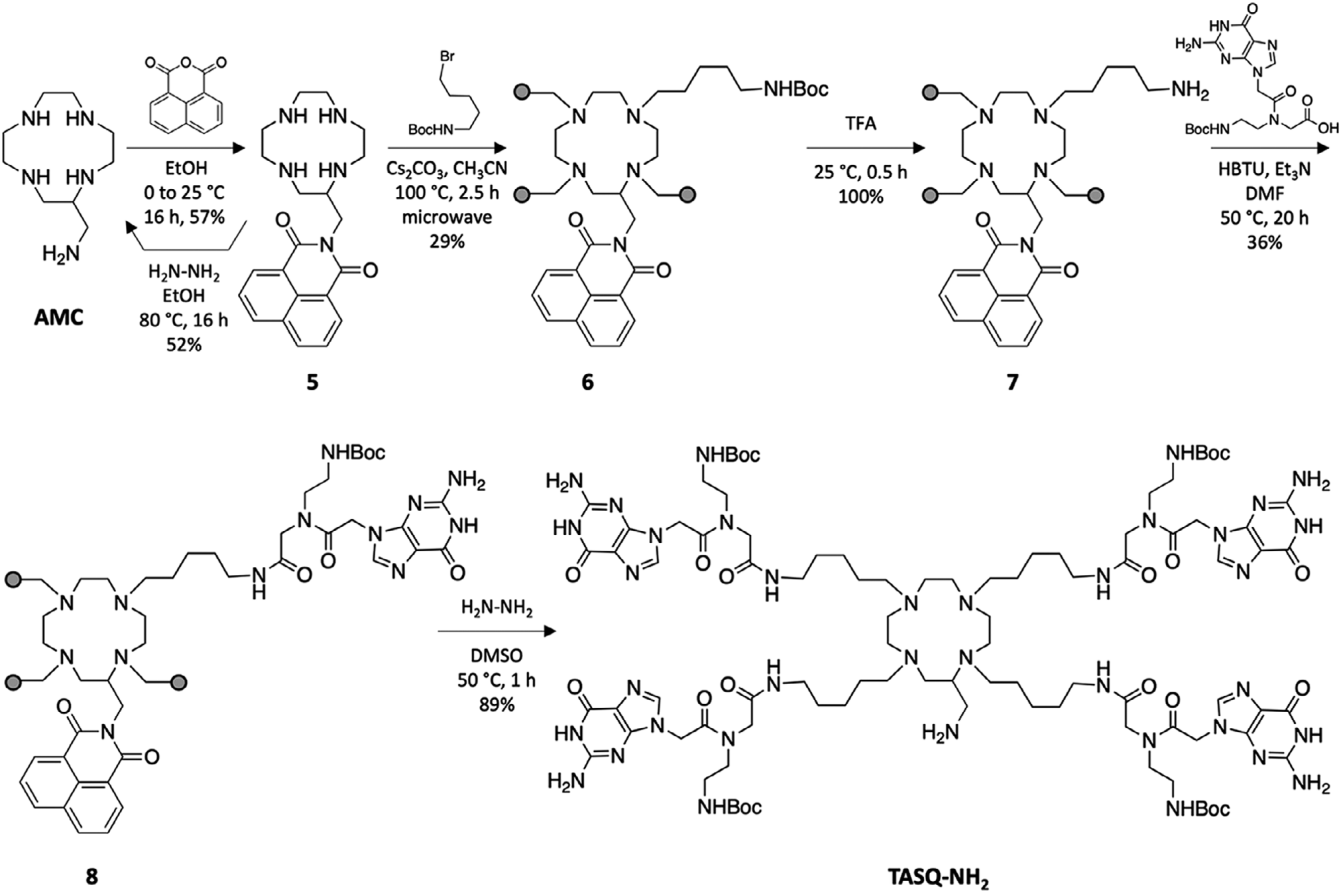
Synthesis of TASQ‐NH_2_, the key intermediate of the convergent synthesis of TASQs (overall chemical yield: 5.3% over 5 steps). The grey dots seen in the structure of **6**, **7,** and **8** indicate that the four arms are identical.

We thus constructed the TASQ scaffold using **5** as the starting material (Scheme 3): the four arms were first introduced using an excess of tert‐butyl‐(5‐bromopentyl)carbamate (8 mol. equiv.), Cs_2_CO_3_ as a base in acetonitrile (50°C, 120 h) to lead to compound **6** in 19% chemical yield. This yield being disappointing, this step was further optimized using microwave activation, which led to obtaining compound **6** in a far shorter time (2.5 h), a simpler purification protocol (see the Experimental part), and a higher chemical yield (29%). After the deprotection of the four primary amines using trifluoroacetic acid (TFA, 25°C, 30 min, quantitative—compound **7**), the amines were coupled with Boc‐^PNA^G‐OH [14] using HBTU (*N,N,N’,N’*‐tetramethyl‐*O*‐(1*H*‐benzotriazol‐1‐yl)uronium hexafluorophosphate) as a coupling agent and Et_3_N as a base in *N,N*‐dimethylformamide (DMF, 50°C, 20 h) to lead to **8** in 36% chemical yield. After the deprotection of the **AMC** sidechain was performed using hydrazine hydrate in dimethylsulfoxide (DMSO, 1h at 50°C), the common intermediate referred to as **TASQ‐NH_2_** was isolated in 89% chemical yield (*ca*. 50‐mg scale). Quite satisfyingly, the naphthalimide protecting group fully meets the expected requirement to withstand both the acidic (TFA) and basic (Cs_2_CO_3_, Et_3_N) steps of this synthesis.

### 2.4 The Convergent Synthesis of TASQs and Their Validation in Vitro

The last part of the convergent synthesis of TASQs requires the functionalization of the primary amine side chain of **TASQ‐NH_2_**. We thus validated the reliability of this novel approach synthesizing **BioCyTASQ**, **MultiTASQ**, **^az^MultiTASQ,** and **photoMultiTASQ** from **TASQ‐NH_2_**. We decided to select HBTU or TSTU (*N,N,N’,N’*‐tetramethyl‐*O*‐(*N*‐succinimidyl)uronium tetrafluoroborate) as coupling agents, the latter uniquely allowing for monitoring by HPLC the in situ formation of the activated esters from biotin, 5‐hexynoic acid, 6‐azido‐hexanoic acid, and 3‐(3‐(but‐3‐yn‐1‐yl)‐3H‐diazirin‐3‐yl)propanoic acid. These syntheses were performed using TSTU or HBTU and DIPEA (*N,N*‐diisopropylethylamine) as a base in dry DMF.

**FIGURE 2:**
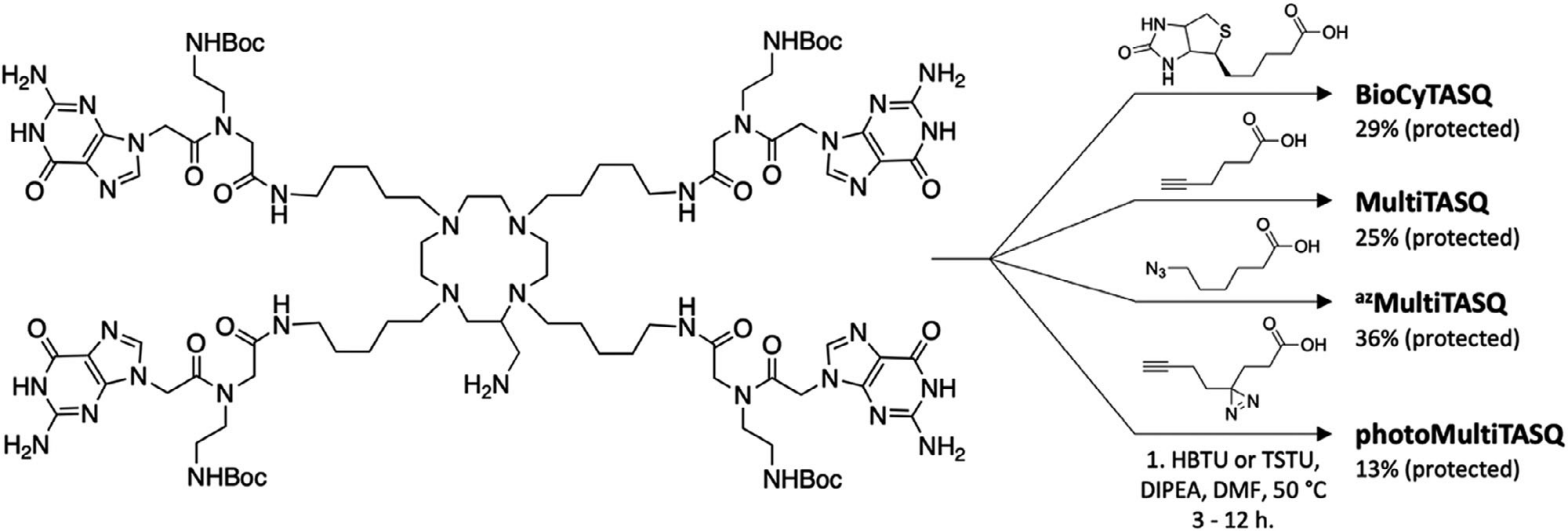
Synthesis of four multivalent TASQs from TASQ‐NH_2_.

As seen in Figure 2, the multivalent TASQs were obtained with low to moderate chemical yield (13 ‐ 36%), which might originate in the rather low reactivity of this primary amine, as the chemical yields obtained during the functionalization of the **AMC** at the very first step of the linear syntheses were in a similar range (21 ‐ 39%, Scheme 1). Several optimizations are currently being investigated to improve this step, which turns out to be particularly difficult with the diazirine‐containing appendage. Among the available options, a systematic screening of the coupling agents (such as hexafluorophosphate azabenzotriazole tetramethyl uronium (HATU) or benzotriazol‐1‐yloxytripyrrolidinophosphonium hexafluorophosphate (PyBOP)) and of the bases used (such as Et_3_N or *N*‐methylmorpholine) must now be performed, along with a focus on the reactant itself (including an optimization of both the stoichiometry and the pace of addition and/or the isolation and characterization of its activated ester, for instance) and on a possible microwave‐assisted activation of this chemical step. We do believe that these efforts will pay off soon; however, obtaining these four protected TASQs (their long‐time storage form), even with yet unoptimized yields, fully validates the strategy described herein, which turns out to be more economically (notably for what concerns the synthesis of **photoMultiTASQ**) and experimentally relevant than the previously developed ones.

## 3 Conclusion

Both the chemical biology and the chemical genetics terms have been used by Linus Pauling in the early 1950s [36]. Since then, their definition has kept evolving with the technological advancements of both chemistry and biology fields [37, 38]. A now accepted definition of chemical biology and chemical genetics could be defined as using chemicals as reversible, positive, and/or negative modulators of either biological systems (chemical biology) or genetic transactions (chemical genetics). The G4 field thus belongs to the latter [39, 40], and the efforts made over the past years have led to the development of ligands to uncover the cellular pathways in which G4s are involved [41]. This has led to strategies aimed at gaining control over the discovered G4‐involving pathways, allowing for a series of breakthroughs in oncology [42, 43], neurology [44, 45] and virology [46].

Our contribution was to develop G4‐interacting molecules as responsive molecular tools able to highlight whether, when and where DNA and RNA G4s fold (and unfold) in cells, along with the modulation of the G4 landscape upon pharmacological treatments. For making a coherent toolbox, we opted for a series of compounds that interact similarly with G4s while displaying different functionalities: the design of TASQs fully meets these specifications since one side of the TASQs (the synthetic G‐quartet) binds G4s efficiently while the other side (the cyclen template) is left free to be modified with an appendage that allows for introducing an additional functionality (i.e., a clickable moiety, a photoactivatable handle, etc.) (Figure 1).

The multivalent TASQs recently developed, that is, **BioCyTASQ**, **MultiTASQ**, **^az^MultiTASQ,** and **photoMultiTASQ**, turned out to be useful to investigate the biology of both DNA and RNA G4s in detail [39, 40, 47], allowing for visualizing (pre‐targeted and in situ click imaging) and identifying them (G4omics) from human cells. However, the way these TASQs were synthesized was not satisfactory, neither in terms of strategy (being linear in nature) nor efficiency (with an overall chemical yield < 3%). We report herein on our effort to simplify the synthetic access to these TASQs, making it convergent thanks to the key common intermediate **TASQ‐NH_2_** which occupies a central place in the latest steps of the TASQs’ synthesis. Thanks to the astute selection of a naphthalimide protecting group for the primary amine sidechain of the **AMC**, the synthesis of **TASQ‐NH_2_** could be scaled up (for instance, **TASQ‐NH_2_** was produced at a 50‐mg scale), and that of multivalent TASQs decided at will thanks to a single additional step.

Admittedly, the overall chemical yields of these syntheses are still low (< 3%); however, their modularity, which allows for the preparation of the different components independently, makes them robust enough to enable the sustainable production of multivalent TASQs. This strategy also opens up brand new possibilities for the design of ever smarter G4 molecular tools, making it possible to introduce, by the end of the synthesis sequence of novel, highly technical and/or sensitive functional handles.

## 4 Experimental Part

### 4.1 AMC Synthesis (Optimized From [29])

Step 1: triethylenetetramine (TETA, 419 g, 60% purity) was dissolved in toluene (800 mL) and cooled to 0°C with an ice bath. The solution was vigorously stirred, and H_2_O (115 mL) was slowly added, monitoring the temperature to avoid excessive heating (< 36°C). A fine precipitate was formed and filtered out. The solid was then dissolved in MeOH, cooled with an ice bath, and MgSO_4_ was added to remove traces of water. After filtration and drying, TETA was obtained as a colorless oil (140 g, extraction yield 55 %). ^1^H NMR: (500 MHz, D_2_O) δ 2.74 – 2.68 (m, 8H), 2.64 (t, J = 6.2 Hz, 4H).

Step 2: The recrystallized TETA (140 g, 0.96 mol) was dissolved in CH_3_CN (5 L), to which Ca(OH)_2_ (142 g, 1.92 mol) was added, and the solution was cooled to 0°C. 2,3‐butanedione (84 mL, 0.96 mol) was added dropwise to the suspension over 2 h. After an additional 20 min of stirring, the suspension was filtered, and the reaction mixture containing compound 1 was used in the next synthetic step without purification. The solution was cooled back to 0°C and 1*H*‐Benzotriazole (Bt, 114 g, 0.96 mol) and K_2_CO_3_ (65 g, 1.92 mol) were added. Separately, chloroacetaldehyde (150 mL, 0.96 mol, 55% v/v in H_2_O) was cooled with an ice bath, and MgSO_4_ was added to remove traces of H_2_O. After filtration, it was added dropwise to the CH_3_CN solution containing compound 1, and the reaction was stirred for 2 h at 0°C. Compound 2 was not isolated; indeed, sodium cyanide (47 g, 0.96 mol) was carefully added, and the reaction mixture was stirred overnight at 25°C. The suspension was then filtered (on celite), and the solvent was evaporated *in vacuo*. The resulting solid was first taken up in CHCl_3_ (600 mL) and vigorously stirred; the suspension was then filtered on celite. After evaporation of the solvent, the same procedure was repeated with Et_2_O (2 L). The organic phases were combined and evaporated *in vacuo*. The resulting oil was dissolved in CHCl_3_ and adsorbed on alumina (3 g of alumina per 1 g of crude material). A Soxhlet extraction was performed with pentane for 42 h, which led, after evaporation of the solvent, to compound 3 in 38% chemical yield (6.8 g, 0.027 mol). ^1^H NMR: (500 MHz, CDCl_3_) δ 4.22 (dd, J = 8.1, 6.0 Hz, 1H), 3.56 – 3.37 (m, 3H), 3.23 – 2.49 (m, 26H), 1.33 (s, 3H), 1.18 – 1.00 (m, 9H). ^13^C NMR: (126 MHz, CDCl_3_) δ 120.4, 119.7, 80.0, 79.5, 78.3, 78.0, 56.5, 55.7, 51.1, 50.8, 50.4, 50.2, 49.8, 49.7, 47.9, 47.4, 46.5, 45.8, 44.3, 43.8, 42.9, 42.6, 16.4, 14.4, 14.1, 13.9.

Step 3: Anhydrous THF was added to a three‐neck round‐bottom flask and cooled at ‐78°C under an inert atmosphere, to which LiAlH_4_ (72 mL, 0.173 mol, 2.4 M THF solution) was added. Separately, compound 3 (21.4 g, 0.086 mol) was dissolved in anhydrous THF (21.3 g, 0.086 mol) and added dropwise to the LiAlH_4_ solution at ‐78°C. The cooling bath was removed, and the reaction mixture was allowed to reach RT overnight, under stirring. The excess of LiAlH_4_ was quenched by cooling the solution again to ‐78°C and gentle addition of H_2_O. The solvent was then evaporated, and the resulting solid was extracted 3 times with CHCl_3_ (3 x 500 mL) and filtered. After evaporation of the solvent *in vacuo*, compound 4 was isolated without further purification (19.9 g, 0.07 mol, 81% chemical yield). ^1^H NMR (500 MHz, CDCl_3_) δ 3.46 – 2.57 (m, 32H), 2.54 – 2.34 (m, 4H), 1.28 – 1.20 (m, 4H), 1.15 (dd, J = 6.5, 2.9 Hz, 4H), 1.12 – 1.08 (m, 4H), 1.08 – 1.04 (m, 4H). ^13^C NMR (126 MHz, CDCl_3_) δ 80.3, 79.4, 78.4, 63.3, 60.4, 52.3, 51.4, 51.3, 50.5, 50.1, 46.4, 46.4, 46.1, 45.6, 45.6, 45.2, 44.80, 44.78, 44.4, 44.3, 43.9, 43.3, 23.0, 16.60, 13.98, 13.90.

Step 4: Compound 4 (19.9 g, 0.07 mol) was dissolved in HCl (46 mL, 37% solution, 0.55 mol) and heated at reflux overnight. The precipitate was filtered and washed with cold EtOH and Et_2_O to obtain AMC as a hydrochloride salt (15.1 g, 0.011 mol, 60% chemical yield). ^1^H NMR (500 MHz, D_2_O) δ 3.50 – 3.03 (m, 16H), 2.92 (m, 1H). ^13^C NMR (126 MHz, D_2_O) δ 51.4, 47.0, 45.6, 44.3, 44.0, 43.9, 42.3, 42.0, 39.3. To obtain unprotonated AMC, the salt was resuspended in a 16 M aqueous solution of NaOH and extracted 3 times with CHCl_3_. The organic phase was then dried with MgSO_4_, filtered, and evaporated *in vacuo* to afford AMC as an oil (quantitative). ^1^H NMR (500 MHz, CDCl_3_) δ 2.75 – 2.41 (m, 17H), 2.01 (m, 6H). ^13^C NMR (126 MHz, CDCl_3_) δ 57.2, 49.3, 48.9, 47.4, 46.7, 46.3, 44.2, 44.1, 41.5 (in agreement with the literature).

### 4.2 TASQ‐NH_2_ Synthesis

Step 1: The deprotonated AMC (20.0 mg, 0.1 mmol, 1.0 equiv.) was dissolved in EtOH (1 mL) at 0°C and 1,8‐naphthalic anhydride (19.7 mg, 0.1 mmol, 1.0 equiv.) was added. The reaction, which slowly returns to RT, was stirred for 16 h under HPLC‐MS monitoring. EtOH was then evaporated, and the crude material was purified by semi‐preparative HPLC (H2O + 0.1% TFA/CH3CN 75:25 to 30:70 over 16 min). The fractions were combined and lyophilized to yield compound 5 as a white solid (21.6 mg, 57% yield). tr_LCMS_ = 2.05 min. MS (ESI+): m/z = 382.3 [M+H]^+^. ESI‐HRMS: *m/z* = 382.22349 [M+H]⁺ (calculated for C_21_H_27_N_5_O_2_: 381.21648. ^1^H NMR (400 MHz, CDCl_3_) δ 8.58 (dd, *J* = 7.3, 1.1 Hz, 2H), 8.20 (dd, *J* = 8.3, 1.1 Hz, 2H), 7.74 (t, *J* = 7.3 Hz, 2H), 4.25 – 4.16 (m, 2H), 3.12 – 3.06 (m, 1H), 2.86 – 2.55 (m, 14H), 2.4 – 2.16 (m, 4H). ^13^C NMR (101 MHz, CDCl_3_) δ 164.72, 134.16, 131.71, 131.53, 128.32, 127.10, 122.63, 53.85, 48.31, 46.99, 46.78, 46.58, 46.44, 46.11, 45.25, 43.31.

Step 2: Compound **5** (123 mg, 0.3 mmol, 1.0 equiv.) was dissolved in anhydrous CH_3_CN (8 mL) in a 25 mL round‐bottom flask. K_2_CO_3_ (356 mg, 2.6 mmol, 8.0 equiv.) and *tert*‐butyl‐N‐(5‐bromopentyl)carbamate (145 mg, 1.3 mmol, 4.1 equiv.) were added to the reaction mixture. The reaction was heated to reflux (70 °C) under HPLC‐MS monitoring. After 120 h, the reaction mixture was cooled to 25°C and the solvent removed *in vacuo*. The crude material was first purified by reverse‐phase flash chromatography (C18 column, H_2_O + 0.1 % TFA / MeCN, gradient 70:30 to 50:50 over 20 min). A second purification was then performed using semi‐preparative HPLC: the compound was dissolved in a 1:1 mixture of H_2_O and CH_3_CN and eluted with a gradient from 35% to 65% over 15 min. The fractions were combined and lyophilized to yield compound **6** (89 mg, 19% yield). Alternatively, compound **6** was synthesized upon microwave activation: to a solution of compound **5** (45.0 mg, 0.12 mmol, 1.0 mol. equiv.) in anhydrous CH_3_CN (2.95 mL) in a microwave‐compatible reaction vial, Cs_2_CO_3_ (307.0 mg, 0.94 mmol, 8.0 mol. equiv.) and *tert*‐butyl‐*N*‐(5‐bromopentyl)carbamate (128.0 mg, 0.48 mmol, 4.1 mol. equiv.) were added. The reaction mixture was stirred for 2 h at 100 °C under microwave irradiation (200 W) and HPLC‐MS monitoring. After 2 h, an additional equivalent of *tert*‐butyl‐*N*‐(5‐bromopentyl)carbamate (31.0 mg, 0.12 mmol, 1.0 mol. equiv.) was added. The mixture was stirred for a further 30 min at 100 °C under microwave irradiation. The reaction mixture was filtered and concentrated under reduced pressure. The crude product was purified directly by semi‐preparative HPLC (H_2_O + 0.1% TFA/CH_3_CN 65:35 to 30:70 over 20 min) to afford compound **6** as a white solid (38 mg, 29%). tr_LCMS_ (aeris): 8.01 min. MS (ESI^+^): *m/z* = 1122.9 [M+H]^+^. ESI‐HRMS: *m/z* = 1122.78899 [M+H]⁺ (calculated for C_61_H_103_N_9_O_10_: 1121.78280). ^1^H NMR (400 MHz, MeOD) δ 8.61 (d, *J* = 8.4 Hz, 2H), 8.42 (dd, *J* = 8.4, 1.2 Hz, 2H), 7.86 (ddd, *J* = 8.2, 7.3, 2.0 Hz, 2H), 4.34 – 4.28 (m, 1H), 3.77 – 3.38 (m, 6H), 3.22 – 2.52 (m, 22H), 1.90 – 1.18 (m, 64H). ^13^C NMR (101 MHz, MeOD) δ 166.04, 158.55, 136.06, 133.29, 132.66, 128.29, 123.38, 79.83, 40.92, 40.70, 30.78, 30.57, 25.33, 25.27, 24.71, 24.58.

Step 3: Compound 6 (148.0 mg, 132 µmol, 1.0 equiv.) was dissolved in an excess of TFA (400 µL) and stirred at 25 °C for 30 min until complete deprotection, as monitored by HPLC‐MS. The reaction mixture was diluted with ultra‐pure water (4 mL) and lyophilized three times to afford compound 7 as a white powder (135.0 mg, quantitative yield). tr_LCMS_ (kinetex) = 0.5 min. MS (ESI⁺): *m/z* = 722.1 [M+H]⁺. ESI‐HRMS: *m/z* = 722.57967 [M+H]⁺ (calculated for C_41_H_71_N_9_O_2_: 721.57308). ^1^H NMR (500 MHz, MeOD) δ 8.62 (d, *J* = 5.0 Hz, 1H), 8.42 (d, *J* = 8.2 Hz, 1H), 7.87 (t, *J* = 7.8 Hz, 1H).), 4.35 – 4.27 (m, 1H), 3.85 – 3.44 (m, 6H), 3.20 – 2.56 (m, 22H), 1.94 – 1.22 (m, 28H). ^13^C NMR (126 MHz, MeOD) δ 164.72, 134.73, 131.91, 131.26, 128.04, 126.92, 121.9, 39.16, 39.10, 38.94, 27.02, 26.74, 23.74, 23.17, 23.07, 21.46.

Step 4: Boc‐^PNA^G‐OH (336.0 mg, 823 µmol, 4.4 equiv.) was dissolved in anhydrous DMF (1 mL). DIPEA (200 µL, 1.12 mmol, 6.0 equiv.) and HBTU (312.0 mg, 823 µmol, 4.4 equiv.) were added sequentially, and the reaction mixture was stirred at 25 °C for 20 min to generate the activated ester. Separately, compound 7 (135.0 mg, 187 µmol, 1.0 equiv.) was dissolved in 1 mL of anhydrous DMF containing DIPEA (200 µL, 1.12 mmol, 6.0 equiv.). The activated ester solution was then added dropwise to the solution of 7, and the mixture was stirred at 50 °C for 22 h under HPLC‐MS monitoring. The reaction mixture was concentrated under reduced pressure, and the crude product was purified by semi‐preparative HPLC (H_2_O + 0.1% TFA / MeCN, gradient 72:28 to 55:45 over 20 min) to afford compound 8 as a white powder (125.0 mg, 36% yield). tr_LCMS_ (aeris) = 5.7 min. MS (ESI⁺): *m/z* = 1144.6 [M+2H]^2^⁺. ESI‐HRMS: *m/z* = 1144.61544 [M+2H]^2^⁺ (calculated for [C_105_H_157_N_37_O_22_]^2+^: 1144.61632).

Step 5: Compound 8 (40.0 mg, 17.5 µmol, 1.0 equiv.) was dissolved in DMSO (final concentration 0.01 M), and hydrazine hydrate (16.3 µL, 525 µmol, 30 equiv.) was added. The reaction mixture was stirred at 50 °C for 1 h under HPLC‐MS monitoring. The crude mixture was purified by semi‐preparative HPLC (H_2_O + 0.1 % TFA / MeCN, 72:28 for 5 min, then 72:28 to 55:45 over 20 min) to afford TASQ‐NH_2_ as a white powder (33.0 mg, 89% yield). tr_LCMS_: 2.9 min MS (ESI⁺): *m/z* = 1054.3 [M+2H]^2^⁺. ESI‐HRMS: *m/z* = 1054.60483 [M+2H]^2^⁺ (calculated for [C_93_H_152_N_37_O_20_]^2+^: 1054.60576).

### 4.3 TASQ Synthesis

A solution of biotin in dry DMF (4.7 µL, 0.6 M, 42.7 µmol, 10.0 mol. equiv.) was prepared. DIPEA (5.0 µL, 51.2 µmol, 12.0 mol. equiv.) and TSTU (15.4 mg, 51.2 µmol, 12.0 mol. equiv.) were added successively. The reaction mixture was stirred for 20 min at 25°C to generate the activated ester, monitored by HPLC. The activated ester was then added to a solution of **TASQ‐NH_2_** in anhydrous DMF (9 mg, 0.2 M, 4.3 µmol, 1 mol. equiv.) at 50°C for 4 h. The reaction mixture was concentrated under reduced pressure, and the crude product was purified by reverse‐phase chromatography (H_2_O + 0.1 % TFA / MeCN, from 72:28 to 45:55 over 20 min) to afford protected **BioCyTASQ** as a white powder (3.2 mg, 29.0 %). tr_LCMS_ (kinetex): 3.15 min. MS (ESI⁺): *m/z* = 1168.2 [M+2H]^2^⁺. ESI‐HRMS: *m/z* = 1178.63633 [M+H+Na]^2^⁺ (calculated for [C_103_H_166_N_39_O_22_SNa]^2^⁺: 1178.63519).

A solution of 5‐hexenoic acid in dry DMF (4.7 µL, 0.6 M, 42.7 µmol, 10.0 mol. equiv.) was prepared. DIPEA (5.0 µL, 51.2 µmol, 12.0 mol. equiv.) and TSTU (15.4 mg, 51.2 µmol, 12.0 mol. equiv.) were added successively. The reaction mixture was stirred for 20 min at 25°C to generate the activated ester, monitored by HPLC. The activated ester was then added to a solution of TASQ‐NH_2_ in anhydrous DMF (9 mg, 0.2 M, 4.3 µmol, 1 mol. equiv.) at 50°C for 5 h. The reaction mixture was concentrated under reduced pressure, and the crude product was purified by reverse‐phase chromatography (H_2_O + 0.1 % TFA / MeCN, from 72:28 to 45:55 over 20 min) to afford protected MultiTASQ as a white powder (2.2 mg, 25.0 %). tr_LCMS_ (kinetex): 3.20 min. MS (ESI⁺): *m/z* = 1102.3 [M+2H]^2^⁺. ESI‐HRMS: *m/z* = 1101.62541 [M+2H]^2+^ (calculated for [C_99_H_159_N_37_O_21_]^2+^: 1101.7995).

A solution of 6‐azidohexanoic acid in anhydrous DMF (8.4 µL, 573.2 µmol, 7.1 mol. equiv.) was prepared. DIPEA (16 µL, 0.8 M, 92.8 mmol, 11 mol. equiv.) and HBTU (21.7 mg, 573.2 µmol, 7.1 mol. equiv.) were added successively. The reaction mixture was stirred for 20 min at 25°C to generate the activated ester, monitored by HPLC. The resulting activated ester was then added to a solution of TASQ‐NH_2_ in anhydrous DMF (17 mg, 0.2 M, 80.7 µmol, 1 mol. equiv.) at 50°C. The reaction was monitored by HPLC‐MS. After 6 h, 7.1 mol. equiv. of the activated ester were added, and the mixture was stirred for a total of 12 h. The reaction mixture was concentrated under reduced pressure, and the crude product was purified by reverse‐phase chromatography (H_2_O + 0.1% TFA / MeCN, from 72:28 to 45:55 over 20 min) to afford protected ^az^MultiTASQ as a white powder (6.5 mg, 36.0%). tr_LCMS_ (kinetex): 3.22 min. MS (ESI⁺): *m/z* = 1124.7 [M+2H]^2^⁺. ESI‐HRMS: *m/z* = 1124.14250 [M+2H]^2^⁺ (calculated for [C_99_H_162_N_40_O_21_]^2+^: 1124.14304).

A solution of 3‐(3‐but‐3‐yn‐1‐yl)‐3H‐diazirin‐3‐yl)propanoic acid in dry DMF (8.0 mg, 0.6 M, 42.7 µmol, 10.0 mol. equiv.) was prepared. DIPEA (5.0 µL, 51.2 µmol, 12.0 mol. equiv.) and TSTU (15.4 mg, 51.2 µmol, 12.0 mol. equiv.) were added successively. The reaction mixture was stirred for 20 min at 25°C to generate the activated ester, monitored by HPLC. The activated ester was then added to a solution of TASQ‐NH_2_ in anhydrous DMF (9 mg, 0.2 M, 4.3 µmol, 1 mol. equiv.) at 50°C for 3 h. The reaction mixture was concentrated under reduced pressure, and the crude product was purified by reverse‐phase chromatography (H_2_O + 0.1 % TFA / MeCN, from 72:28 to 45:55 over 20 min) to afford protected photoMultiTASQ as a white powder (1.1 mg, 13.0%). tr_LCMS_ (kinetex): 3.20 min. MS (ESI⁺): *m/z* = 1128.8 [M+2H]^2^⁺. ESI‐HRMS: *m/z* = 1128.13458 [M+2H]^2+^ (calculated for [C_101_H_161_N_39_O_21_]^2^⁺: 1128.136775).

## Supporting Information

Figures S1‐S29: characterizations of compounds **3**‐**8**, **TASQ‐NH_2_** and protected **BioCyTASQ**, **MultiTASQ**, **^az^MultiTASQ** and **photoMultiTASQ** by ^1^H and ^13^C NMR, HPLC, MS and HRMS, and HPLC‐MS.

## Conflicts of Interest

The multivalent TASQs have been patented by the CNRS [48] and some of them (BioCyTASQ and MultiTASQ) licensed to Merck KGgA for commercialization.

## Supporting information




**Supporting File 1**: chem70473‐sup‐0001‐SuppMat.docx

## Data Availability

The data that support the findings of this study are available from the corresponding author upon reasonable request.
