# Supplementary material for: Convergent Synthesis of Template‐Assembled Synthetic G‐Quartets (TASQs) Used as Biomimetic and Multivalent G‐Quadruplex (G4) Ligands
Source: Chemistry. 2025 Nov 20;31(71):e03002. doi: 10.1002/chem.202503002 (PMC12734661; doi:10.1002/chem.202503002)
Supplement: Supplementary file 1 — Supporting File 1: chem70473‐sup‐0001‐SuppMat.docx [file CHEM-31-e03002-s001.pdf]

Supporting information

Convergent Synthesis of Template-Assembled Synthetic G-Quartets (TASQs) Used as Biomimetic and Multivalent G-Quadruplex (G4) Ligands

Sandy Raevens,[a] Cécile Desingle,[a] Marc Pirrotta,[a] Francesco Rota Sperti,[a] Andréa Pieri,[a] Pauline Lejault,[a] Ibai E. Valverde*[a] and David Monchaud*[a]

[a]S. Raevens, C. Desingle, M. Pirrotta, F. Rota Sperti, A. Pieri, Dr. P. Lejault, Dr. I. E. Valverde, Dr. D. Monchaud ICMUB CNRS UMR6302, Université Bourgogne Europe (UBE) Dijon, France, 9, Av. Alain Savary, 21078 Dijon, France. E-mail: ibai.valverde@u-bourgogne.fr; david.monchaud@cnrs.fr

Methods. Chemicals were provided by Merck/Sigma-Aldrich, Alfa Aesar, or Fluorochem with the exception of 3-(3-(but-3-yn-1-yl)-3*H*-diazirin-3-yl)propanoic acid that was purchased from Enamine, TFA that was purchased from Fischer Chemicals and AF594 azide that was purchased from Invitrogen. Solvents were purchased from VWR. Unless noted otherwise, all commercially available reagents and solvents were used without further purification. Dry solvents (HPLC-grade) were dried over alumina cartridges prior use using a solvent purification system PureSolv PS-MD-5 model from Innovative Technology (water content for CH_2Cl_2: 50 ppm; MeCN: 12.5 ppm, determined by Karl Fischer titration). HPLC-grade CH_3CN used for HPLC-MS analyses was obtained from Fischer Chemical. CH_3CN used in semi-preparative RP-HPLC purifications was obtained from VWR (technical). All aq. mobile phases for HPLC were prepared using water purified with a PURELAB Chorus system from ELGA (purified to 18.2 MΩ.cm). Yields were calculated based on isolation of the compounds. Purity was determined from the integration of RP-HPLC-MS chromatograms at 214/280 nm and/or by ^{13}C NMR.

Instruments. ^{1}H NMR and ^{13}C NMR spectra were recorded with a Brüker Avance 500 MHz spectrometer. All coupling constants are measured in hertz (Hz) and chemical shifts (δ) are expressed in parts per million (ppm) using residual signals of partially deuterated solvent signals summarized in 2010 by Fulmer et al. (*Organometallics* **2010**, *29*, 2176). Standard abbreviations indicating multiplicity were used as follows: s = singlet, d = doublet, t =triplet, q = quadruplet, m = multiplet, br = broad. RP-HPLC analyses were performed on a Thermo Scientific Vanquish™ Flex instrument (pump + autosampler at 20°C + column oven at 25°C) equipped with a UV-visible DAD and ISQ-EM single quadrupole mass spectrometer. LRMS analyses were achieved with a Thermo Scientific ISQ-EM single quadrupole mass spectrometer equipped with an electrospray (ESI) source. The following MS source parameters were used in HPLC mode if no further specification is mentioned: ion transfer tube temperature: 320°C, gas flow: sheath 40 / aux 5 / sweep 0, spray Voltage: 3.5 kV, spray current: 0.75 µA, capillary temperature: 275°C, resolution (m/z = 200): 240 000. Mass calibration in the 100-2000 Da mass range was operated using the commercially available

Pierce Flexmix calibration solution (ThermoFisher Scientific, #15988796), every month; a one-point mass calibration (fluoranthene) was operated for control every week.

The columns used for analytical separations were:
- System 1 : Column Phenomenex Kinetex (2.6 µm, C18, 100 Å, 50 × 2.1 mm) with a gradient of 0-100 % B/A over 5 minutes at a 0.5 mL/min flow
- System 2 : Column Phenomenex Aeris (3.6 µm, C18, 200 Å, 150 × 2.1 mm) with a gradient of 5-55% B/A over 8 minutes at a 0.5 mL/min flow

Eluent A: H_2O+0.1% formic acid, and eluent B: CH_3CN+0.1% formic acid.

Purifications by semi-preparative HPLC were performed on a Thermo-Dionex Ultimate 3000 instrument equipped with a RS Variable Detector (four distinct wavelengths). HPLC system was equipped with a Jupiter Proteo 4 µm 90Å column (250 x 21.2 mm, AXIA packed). Purifications by reversed-phase flash chromatography were performed on a PuriFlash 5.250 from Interchim. Products were detected by UV at 214 and 254 nm. Eluents available for purification were water with 0.1 % TFA and acetonitrile. Prepacked FlashPure Silica columns (C18, 40 µm irregular particles, Büchi) were used. High resolution mass spectrometry analyses were recorded on Orbitrap Exploris 240 mass spectrometers (Thermo Scientific) with an ESI ion source. Lyophilization was performed with a Christ Alpha 2-4 LD plus.

Microwave-assisted reactions were carried out using the Discover CEM system. Reactions can be performed over a temperature of 40 to 300 °C and at pressures of up to 30 bar

Analytical data of synthesized compounds

The chemical characterizations of **AMC** are identical to Ref. 19a.

For the sake of simplification compounds **1**, **3** and **4** have been presented as a single isomer. However, it is worth noting that, due to the formation of isomers during the formation of the bis-aminal bridge and the formation of diastereoisomers during the ring-closing step, **1**, **3** and **4** are always isolated as a mixture of isomers (Scheme 1), as can be seen by ^{1}H NMR. After hydrolysis of the bis-aminal, **4** provides the **AMC** as a racemic mixture.

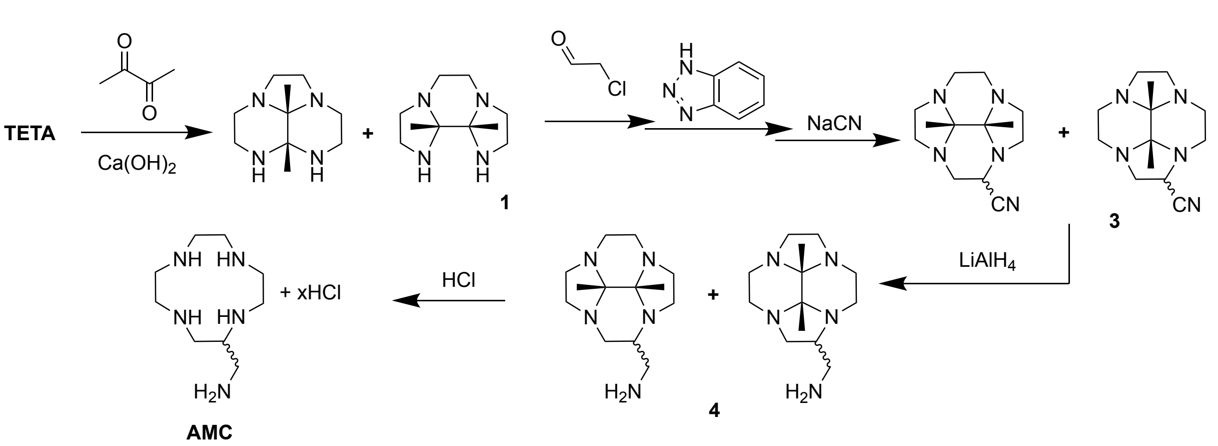

Scheme 1

Recrystallized TETA

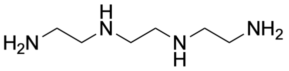

^{1}H NMR: (500 MHz, D_2O) δ 2.73 – 2.66 (m, 8H), 2.62 – 59 (m, 4H).

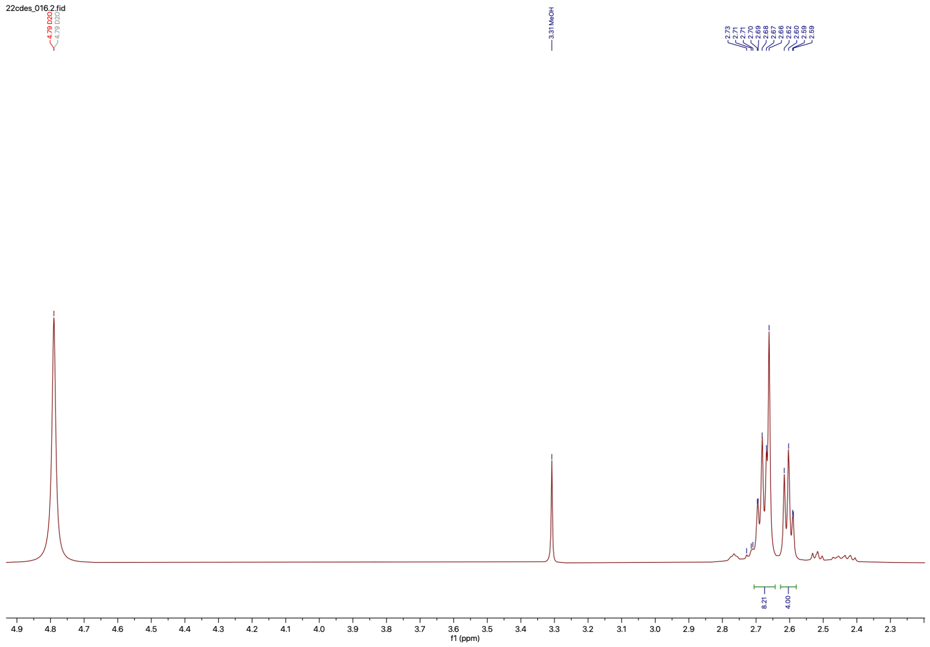

Figure S1: ^{1}H NMR (400 MHz) of compound recrystallized TETA in D_2O

Compound 3

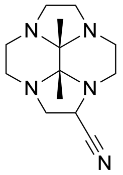

^{1}H NMR: (500 MHz, $CDCl_3$) δ 4.28 (dd, J = 8.1, 6.0 Hz, 1H), 3.72 – 2.05 (m, 81H), 1.39 (s, 3H), 1.14– 1.12 (m, 6H). The ^{1}H NMR spectrum displays a large number of signals due to the presence of a mixture of isomers and diastereoisomers.

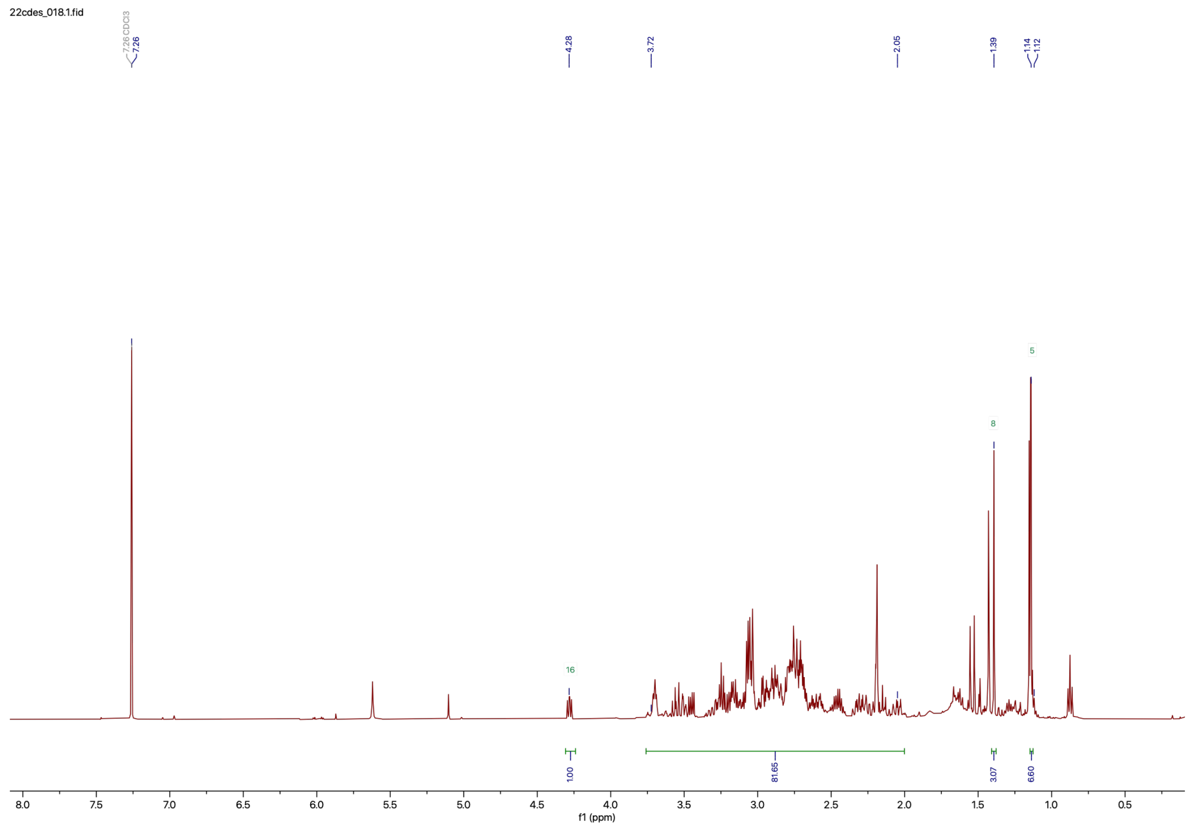

Figure S2: ^{1}H NMR (500 MHz) of compound 3 in $CDCl_3$

Compound 4

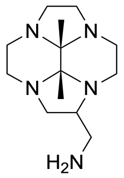

^{1}H NMR (500 MHz, $CDCl_3$) δ 3.36 – 2.60 (m, 32H), 2.60 – 2.38 (m, 6H), 2.00 – 1.80 (m, 12H), 1.36 – 1.15 (m, 8H), 1.15 – 1.05 (m, 4H). The ^{1}H NMR spectrum displays a large number of signals due to the presence of a mixture of isomers and diastereoisomers .

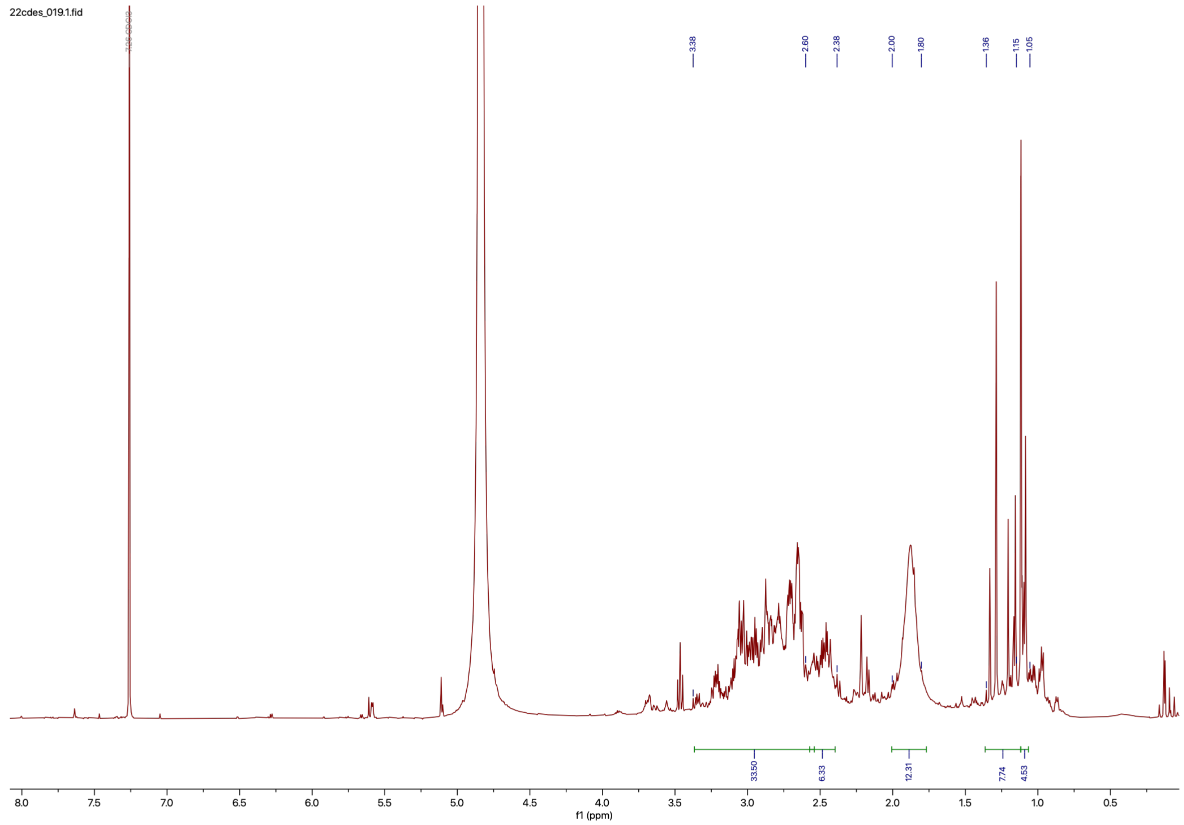

Figure S3: ^{1}H NMR (500 MHz) of compound 4 in $CDCl_3$

AMC

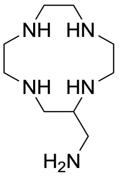

^{1}H NMR (500 MHz, $CDCl_3$) δ 2.78– 2.55 (m, 17H), 1.89 (m, 6H).
^{13}C NMR (126 MHz, $CDCl_3$) δ 57.44, 47.00, 46.87, 46.60, 46.52, 46.31, 46.26, 44.81, 44.31. (in agreement with the literature – Ref. 19a).

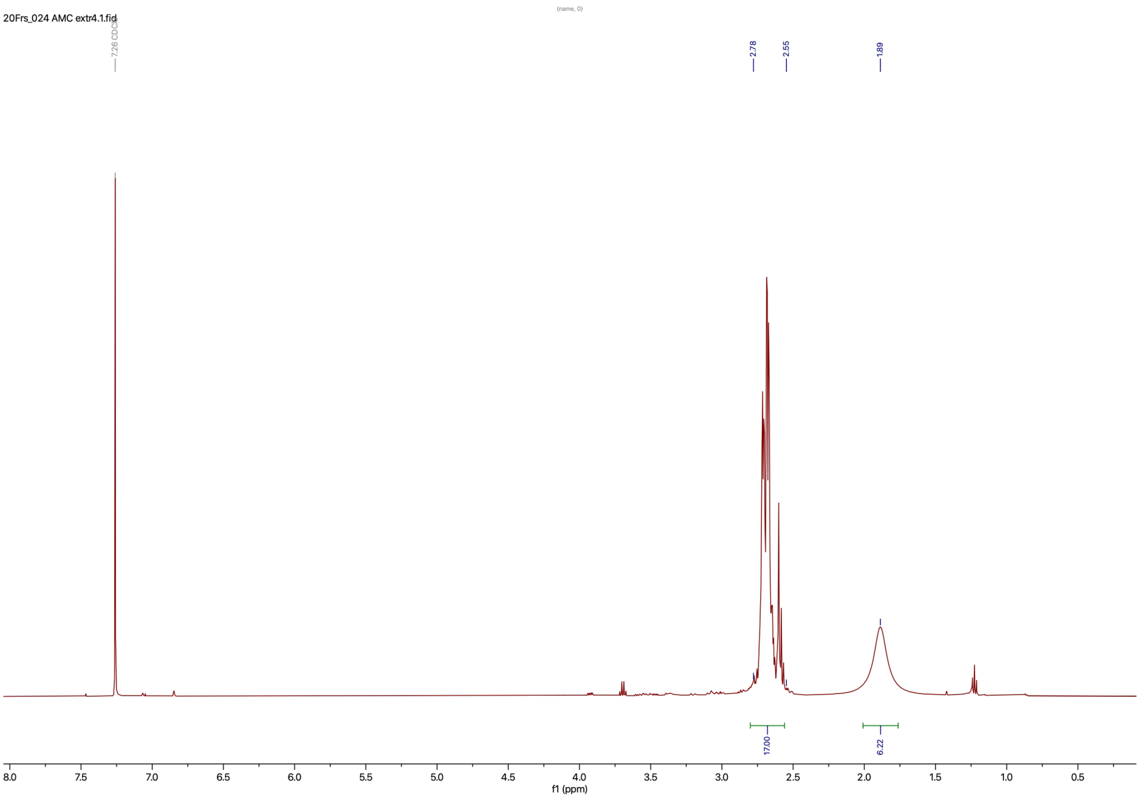

Figure S4: ^{1}H NMR (500 MHz) of compound AMC in $CDCl_3$

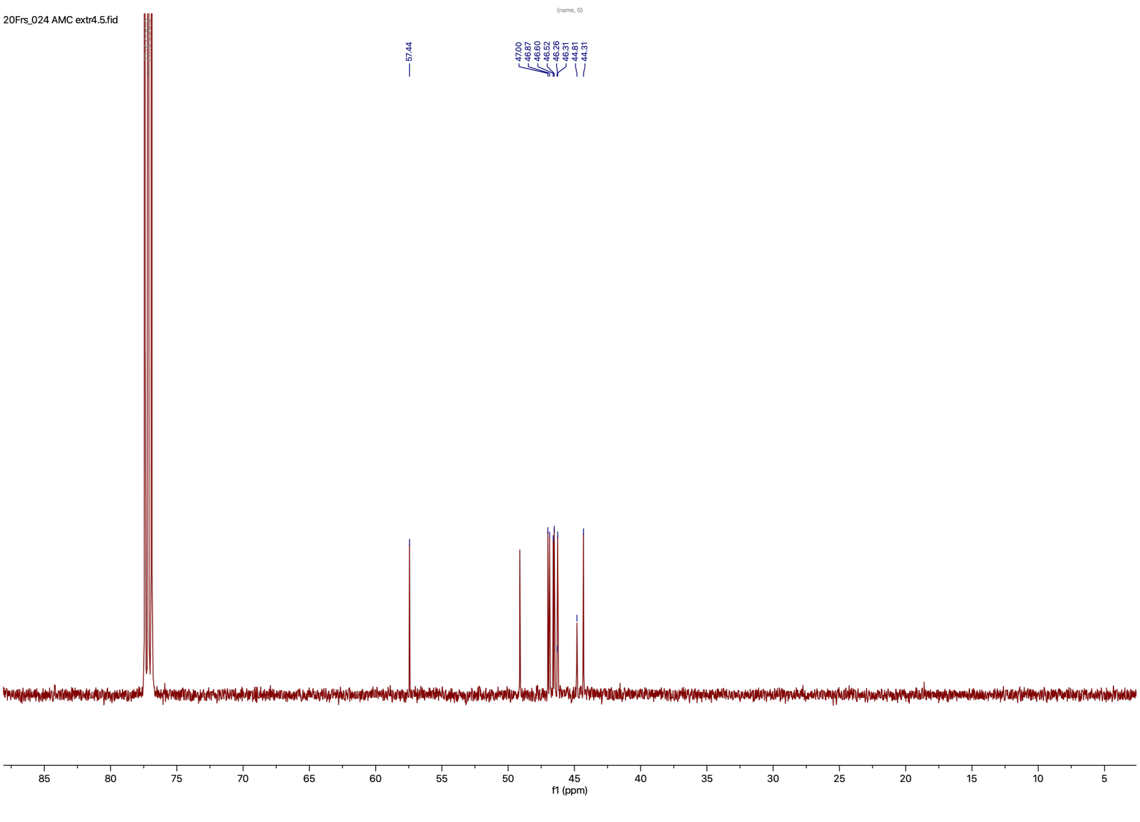

Figure S5: ^{1}H NMR (126MHz) of compound AMC in $CDCl_3$

Compound 5

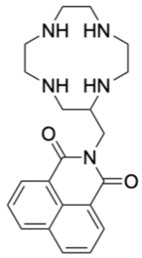

tr_{LCMS} (kinetex) = 2.05 min.
MS (ESI+): m/z = 382.3 $[M+H]^+$**.**
ESI-HRMS: $[M+H]^+$ m/z = 382.22349(calculated for $C_{21}H_{27}N_5O_2$: 381.21648).
^{1}H NMR (400 MHz, $CDCl_3$) δ 8.58 (dd, *J* = 7.3, 1.1 Hz, 2H), 8.20 (dd, *J* = 8.3, 1.1 Hz, 2H), 7.74 (t, *J* = 7.3 Hz, 2H), 4.25 – 4.16 (m, 2H), 3.12 – 3.06 (m, 1H), 2.86 – 2.55 (m, 14H), 2.4 – 2.16 (m, 4H).
^{13}C NMR (101 MHz, $CDCl_3$) δ 164.72, 134.16, 131.71, 131.53, 128.32, 127.10, 122.63, 53.85, 48.31, 46.99, 46.78, 46.58, 46.44, 46.11, 45.25, 43.31.

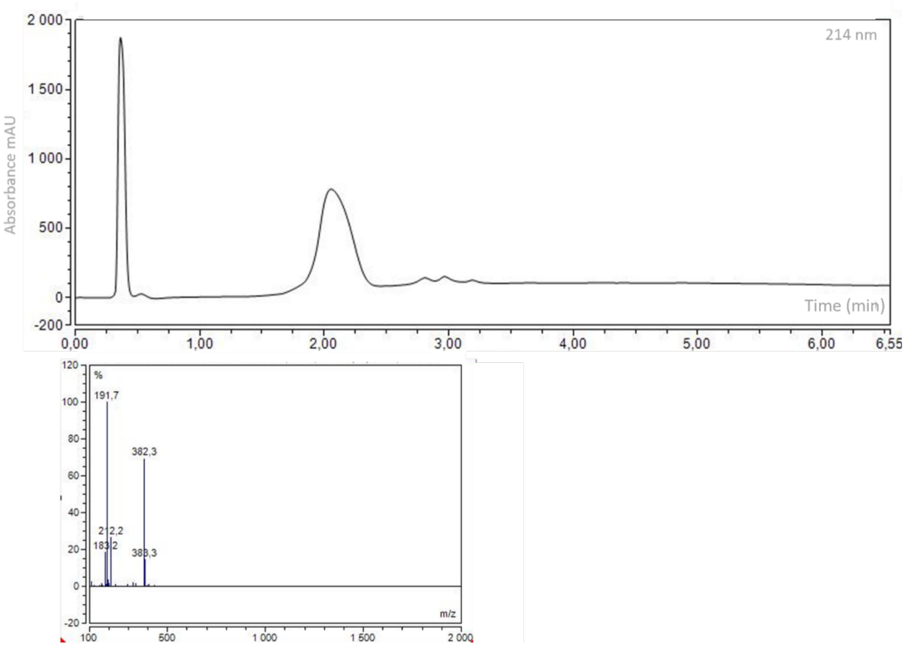

Figure S6: HPLC chromatogram and mass spectrum of 5

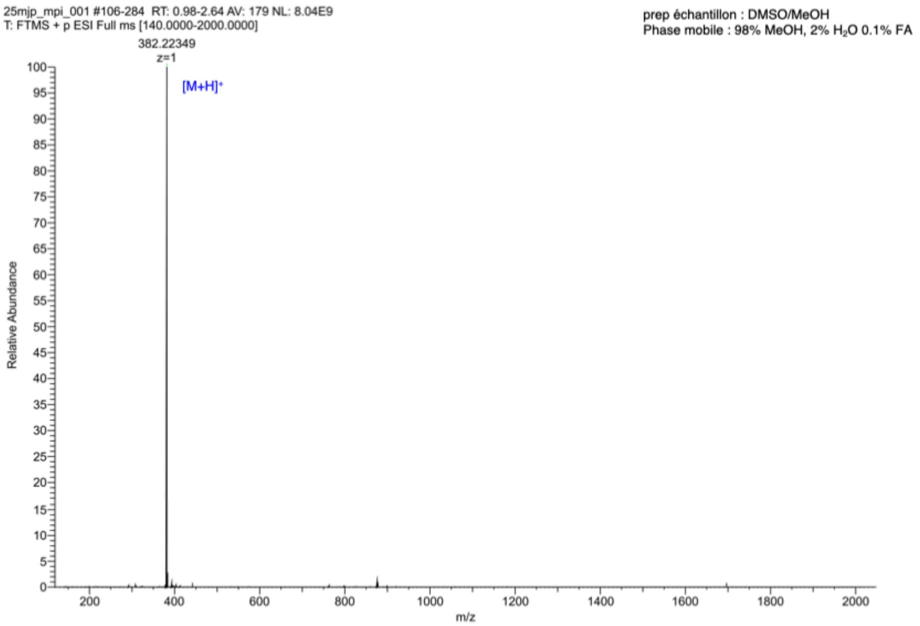

Figure S7 : HRMS of 5

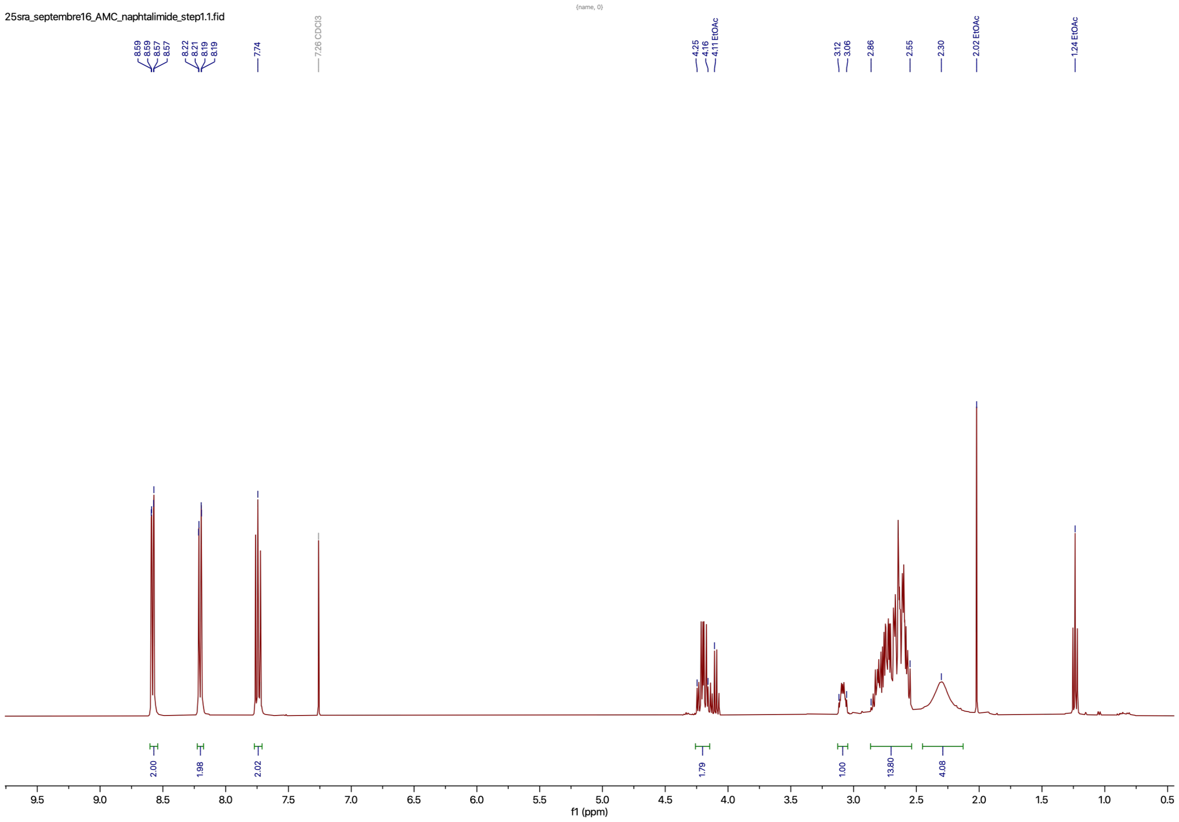

Figure S8: ^{1}H NMR (400 MHz) of compound 5 in $CDCl_3$

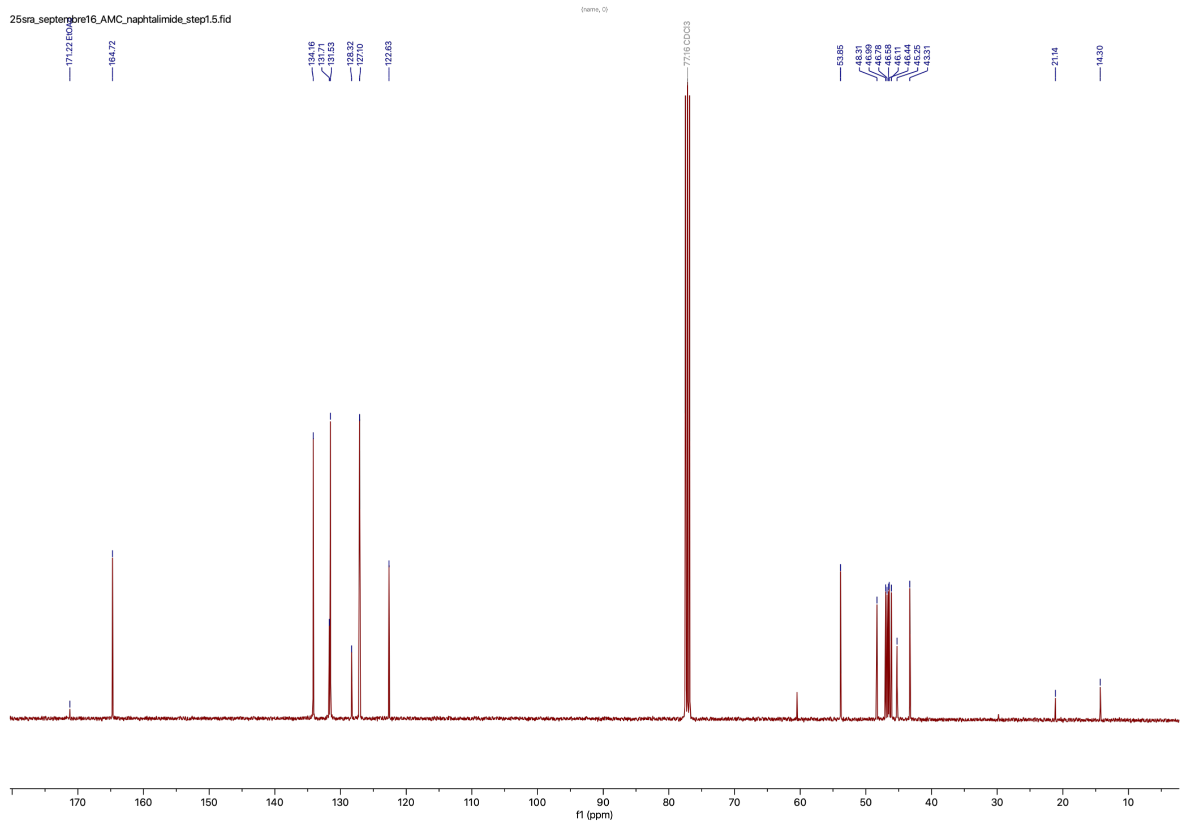

Figure S9: ^{13}C NMR (101 MHz) of compound 5 in $CDCl_3$

Compound 6

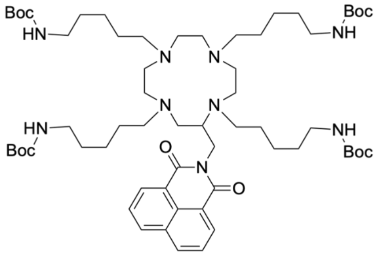

tr$_{LCMS}$ (aeris) : 8.01 min.
MS (ESI+) : [M+H]+ m/z = 1122.9.
ESI-HRMS: [M+H]$^+$ m/z = 1122.78899 (calculated for $C_{61}H_{103}N_9O_{10}$: 1121.78280).
^{1}H NMR (400 MHz, MeOD) δ 8.61 (d, *J* = 8.4 Hz, 2H), 8.42 (dd, *J* = 8.4, 1.2 Hz, 2H), 7.86 (ddd, *J* = 8.2, 7.3, 2.0 Hz, 2H), 4.34 – 4.28 (m, 1H), 3.77 – 3.38 (m, 6H), 3.22 – 2.52 (m, 22H), 1.90 – 1.18 (m,64H).
^{13}C NMR (101 MHz, MeOD) δ 166.04, 158.55, 136.06, 133.29, 132.66, 128.29, 123.38, 79.83, 40.92, 40.70, 30.78, 30.57, 25.33, 25.27, 24.71, 24.58.

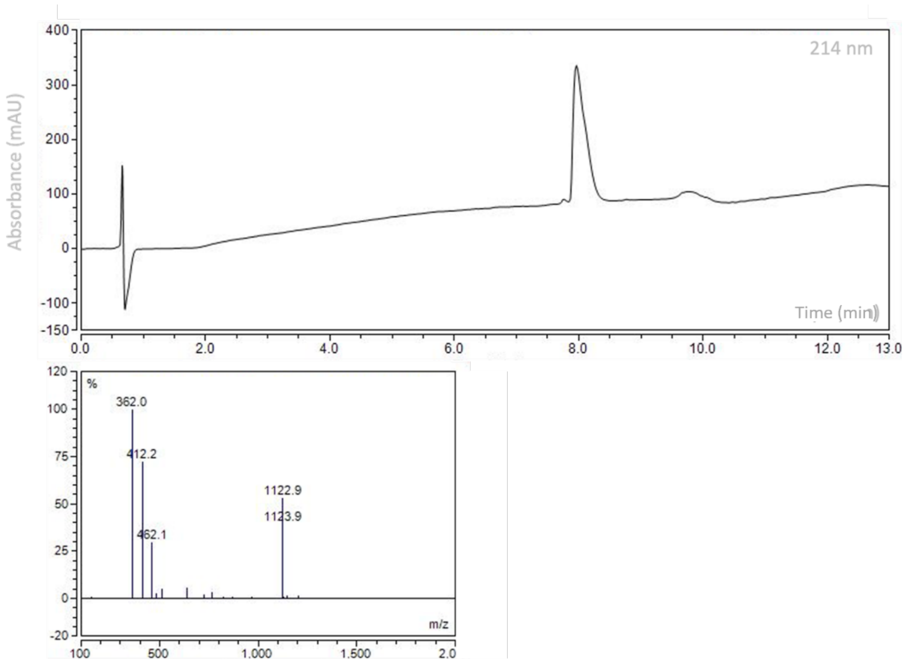

Figure S10: HPLC chromatogram and mass spectrum of 6

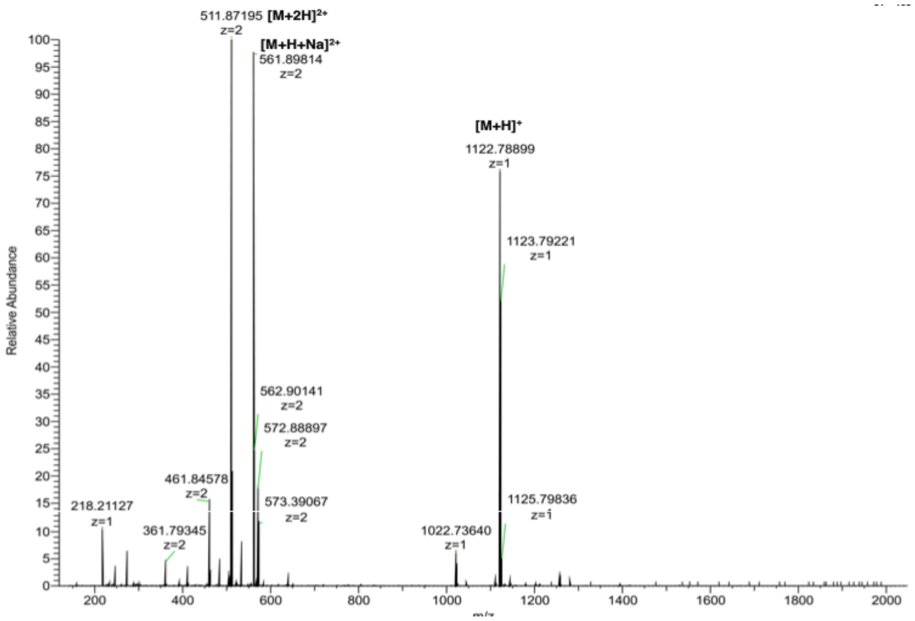

Figure S11 : HPLC-MS of 6

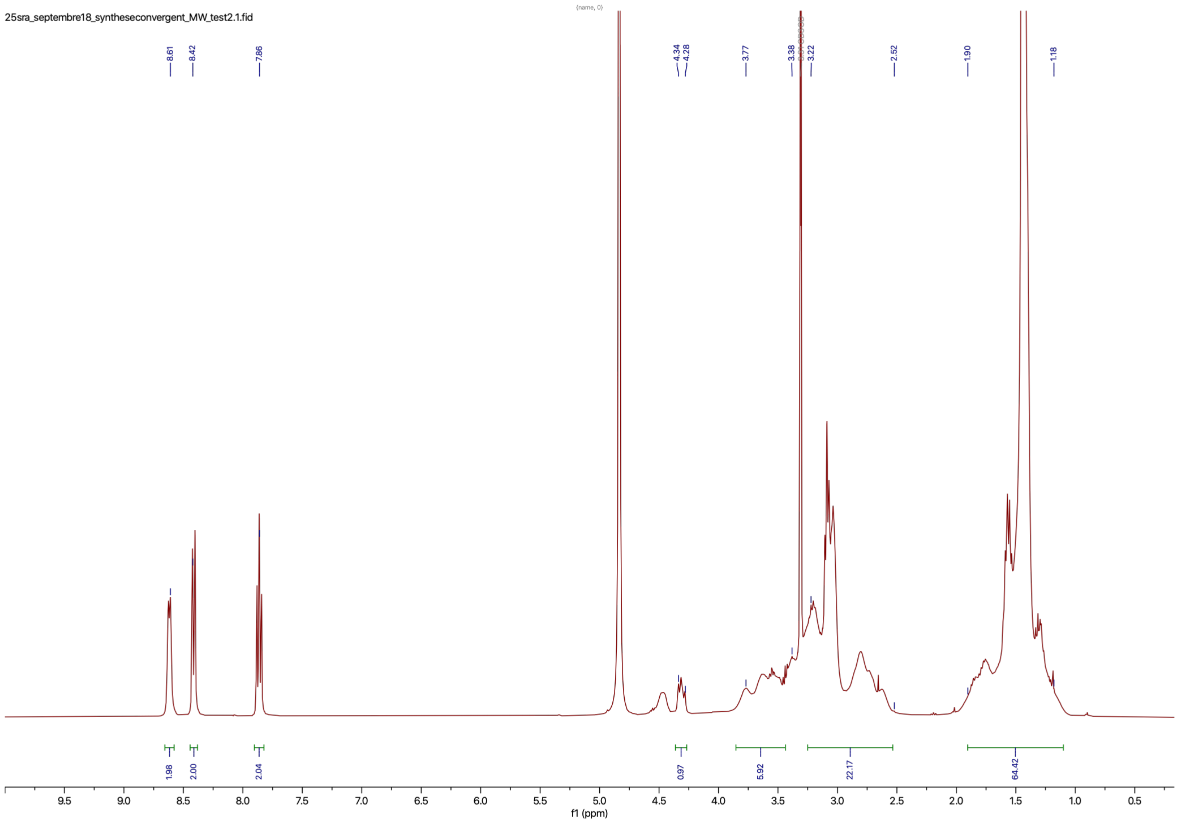

Figure S12: ^{1}H NMR (400 MHz) of compound 6 in MeOD

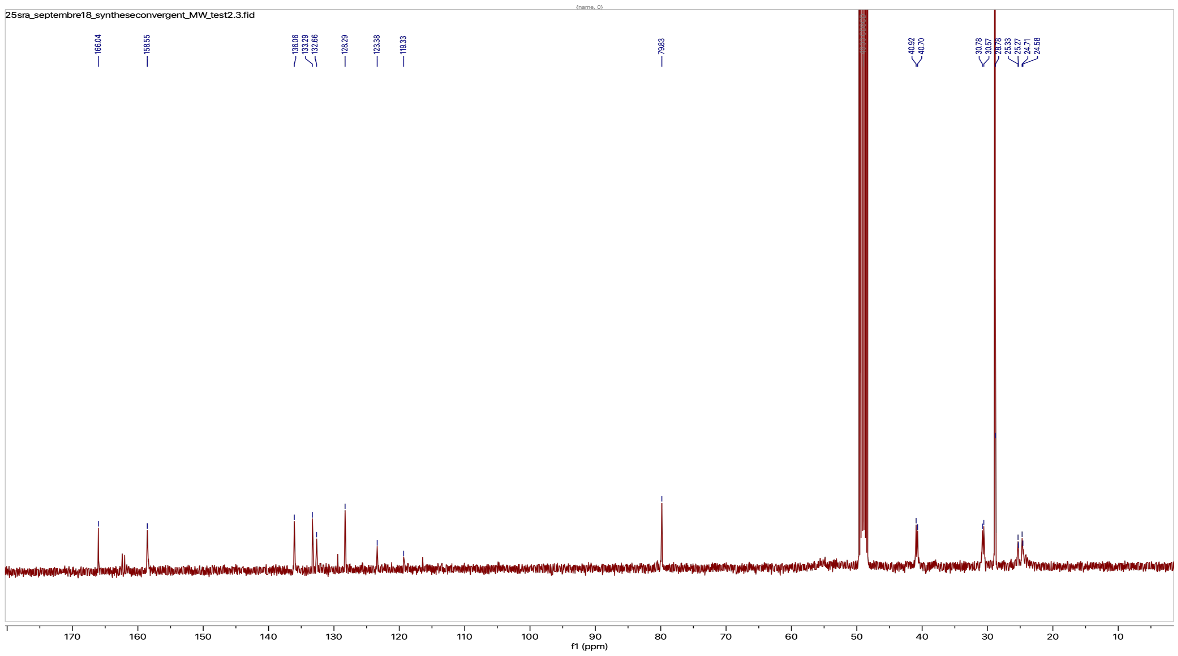

Figure S13: ^{13}C NMR (101 MHz) of compound 6 in MeOD

Compound 7

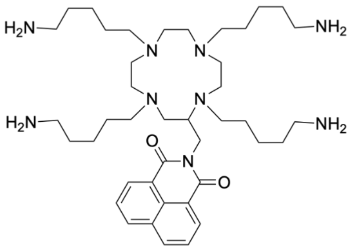

tr$_{LCMS}$ (kinetex) = 0.5 min.
MS (ESI$^+$): m/z = 722.1 [M+H]$^+$.
ESI-HRMS: [M+H]$^+$ m/z = 722.57967 (calculated for $C_{41}H_{71}N_9O_2$: 721.57308).
^{1}H NMR (500 MHz, MeOD) δ 8.62 (d, *J* = 5.0 Hz, 1H), 8.42 (d, *J* = 8.2 Hz, 1H), 7.87 (t, *J* = 7.8 Hz, 1H).), 4.35 – 4.27 (m, 1H), 3.85 – 3.44 (m, 6H), 3.20 – 2.56 (m, 22H), 1.94 – 1.22 (m, 28H).
^{13}C NMR (126 MHz, MeOD) δ 164.72, 134.73, 131.91, 131.26, 128.04, 126.92, 121.9, 39.16, 39.10, 38.94, 27.02, 26.74, 23.74, 23.17, 23.07, 21.46.

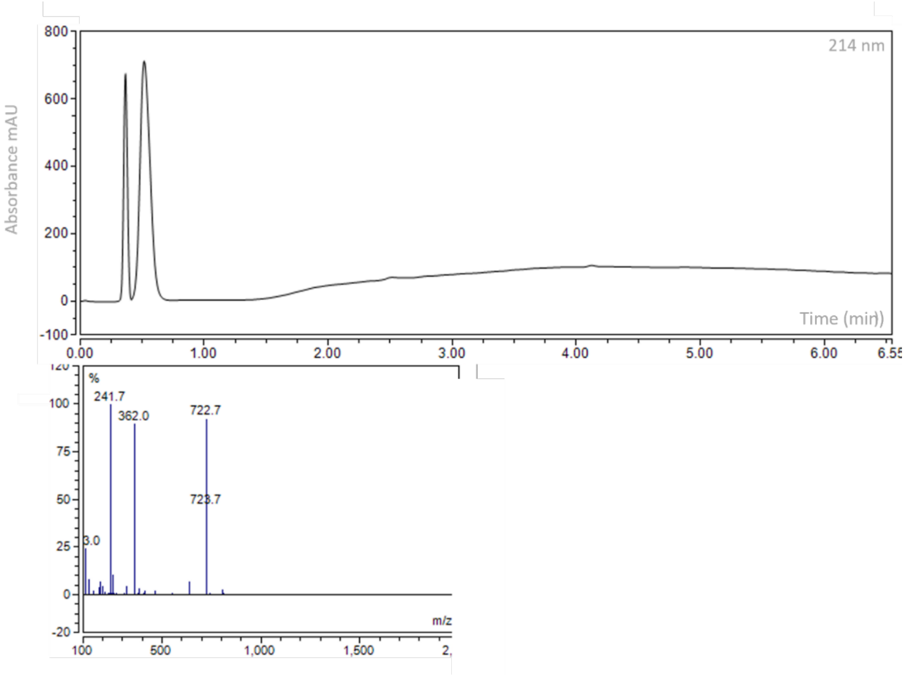

Figure S14: HPLC chromatogram and mass spectrum of 7

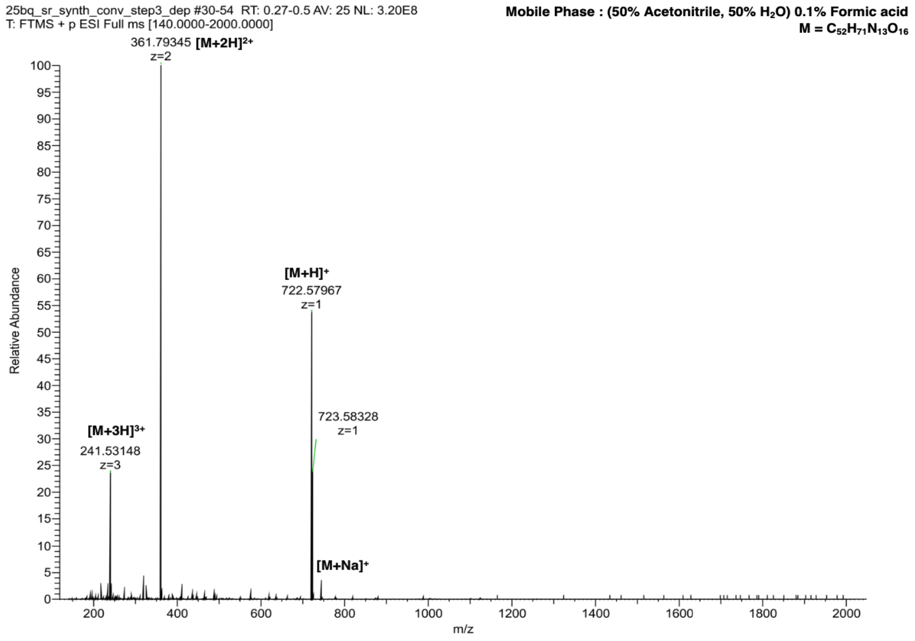

Figure S15: HRMS of 7

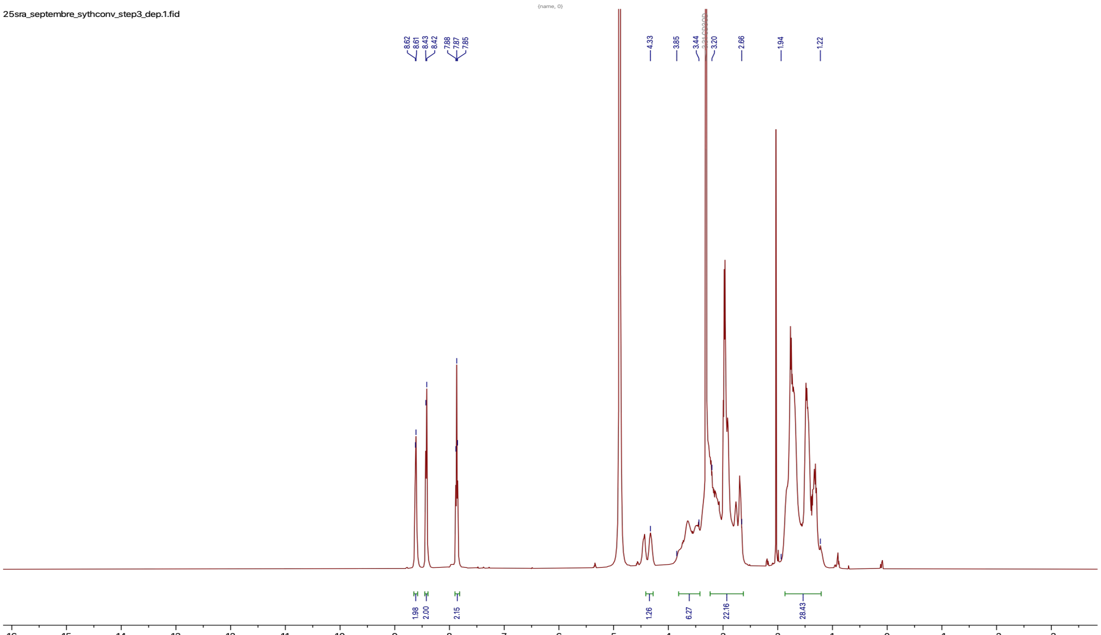

Figure S16: ^{1}H NMR (500 MHz) of compound 7 in MeOD

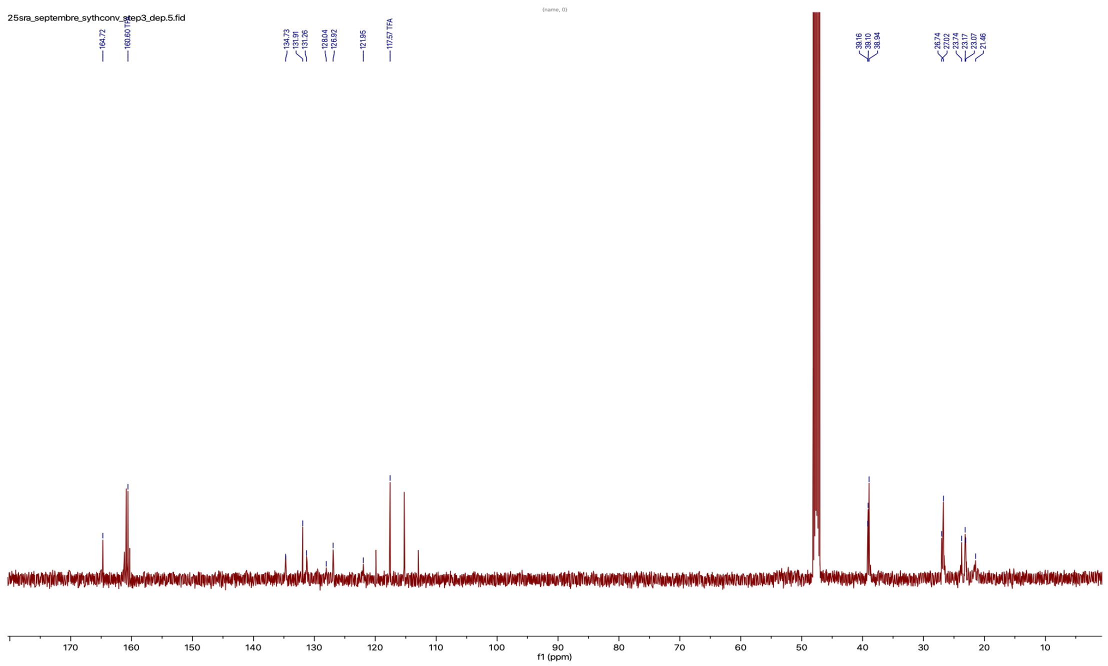

Figure S17: ^{13}C NMR (126 MHz) of compound 7 in MeOD

Compound 8:

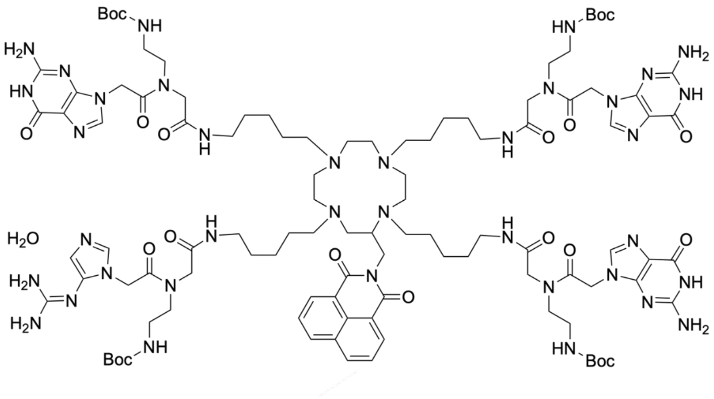

tr$_{LCMS}$ (aeris) = 5.7 min.
MS (ESI$^+$): ***m/z*** = 1144.6 [M+2H]$^{2+}$.
ESI-HRMS: ***m/z*** = 1144.61544 [M+2H]$^{2+}$ (calculated for [$C_{105}H_{157}N_{37}O_{22}$]$^{2+}$: 1144.61632).

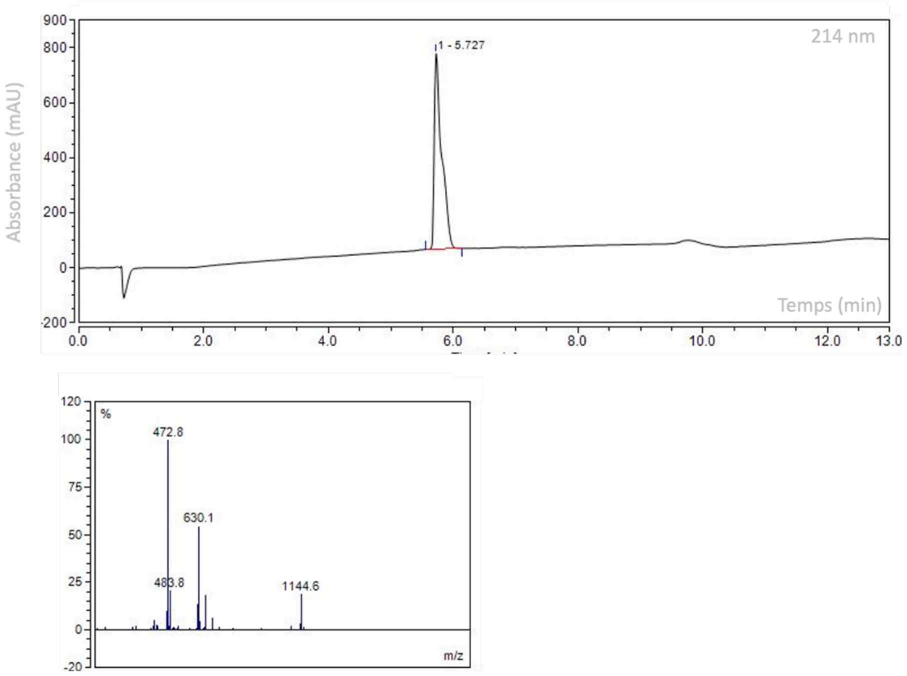

Figure S18 : HPLC chromatogram and mass spectrum of 8

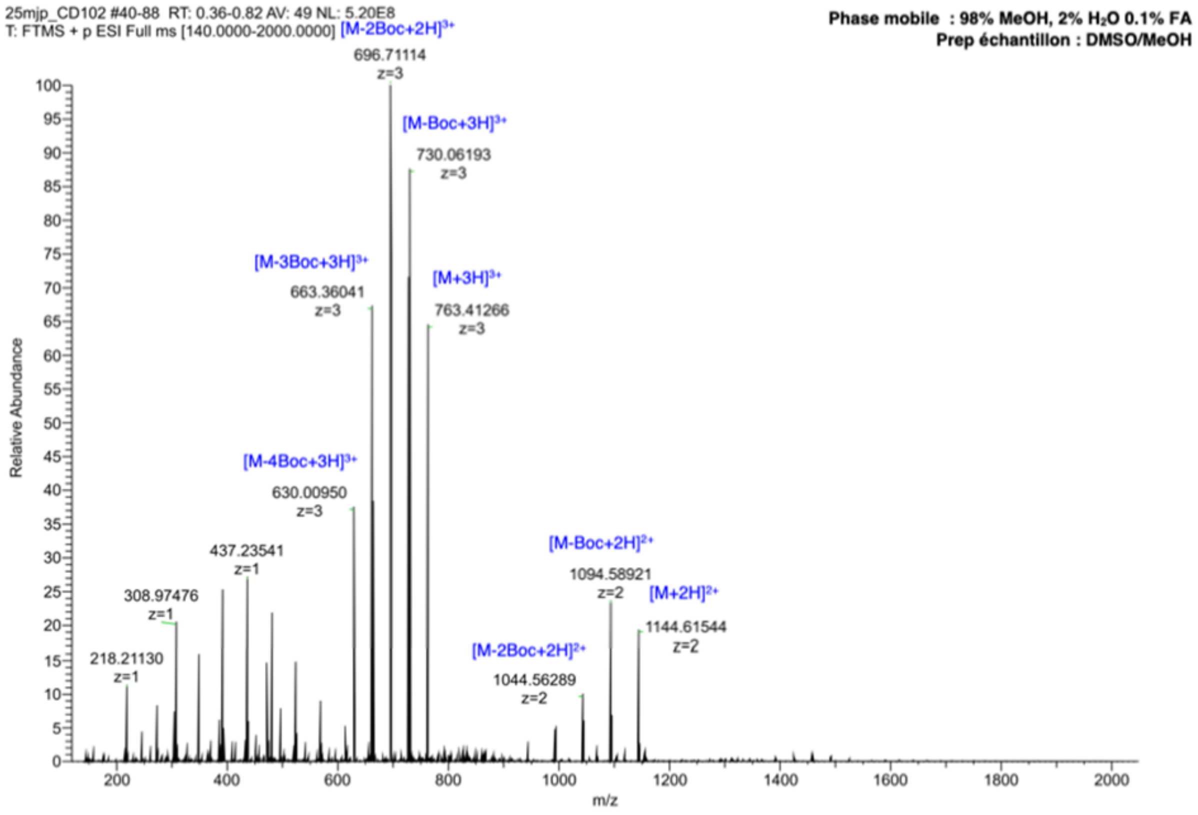

Figure S19: HRMS of 8

TASQ-NH$_2$:

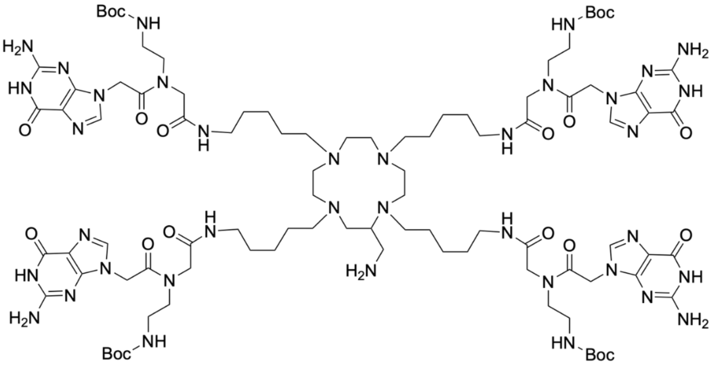

tr$_{LCMS}$ (kinetex): 2.9 min
MS (ESI$^+$): [M+2H]$^{2+}$ m/z = 1054.3
ESI HRMS: [M+2H]$^{2+}$ m/z = 1054.60483 (calculated for $[C_{93}H_{153}N_{37}O_{20}]^{2+}$: 1054.60576).

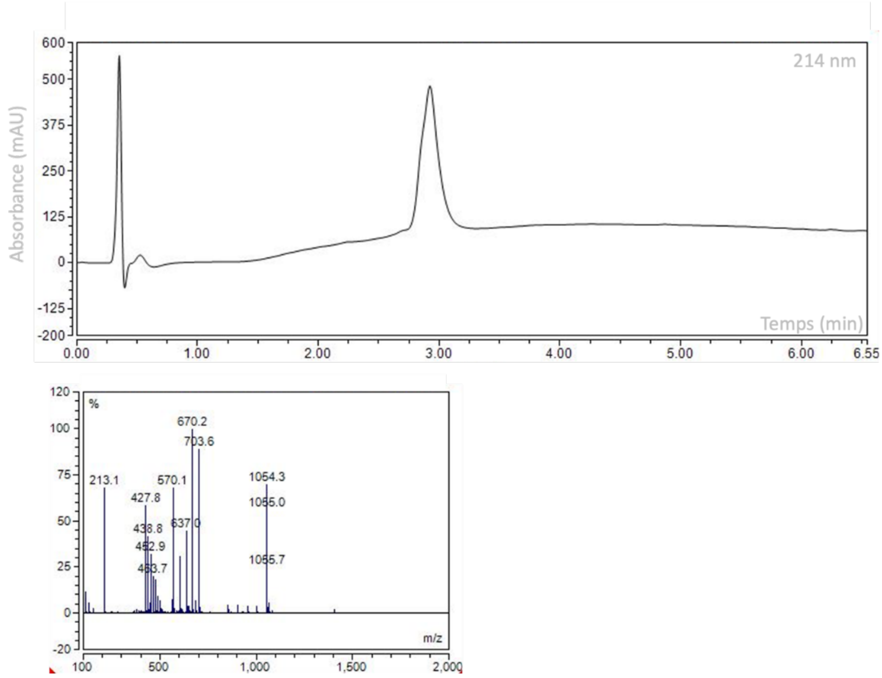

Figure S20: HPLC chromatogram and mass spectrum of TASQ-NH$_2$

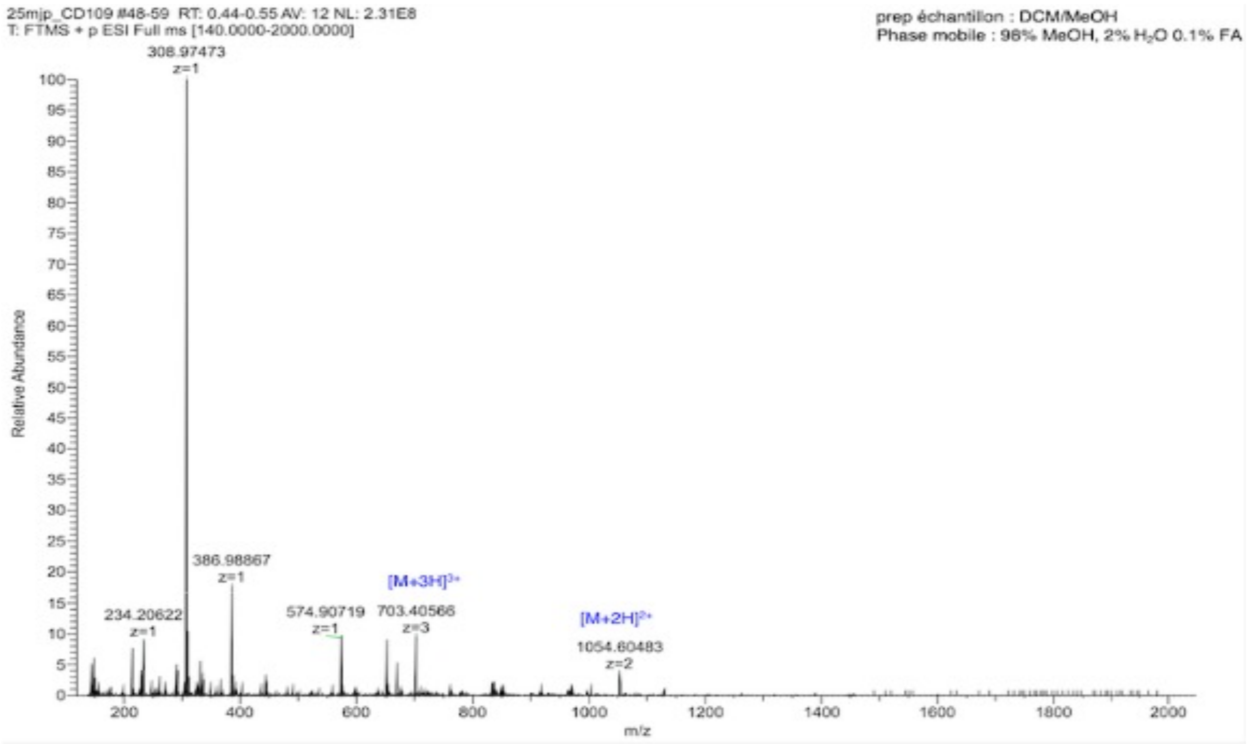

Figure S21: HRMS of TASQ-NH_2

Synthesis of TASQs *via* convergent synthesis

Protected BioCyTASQ

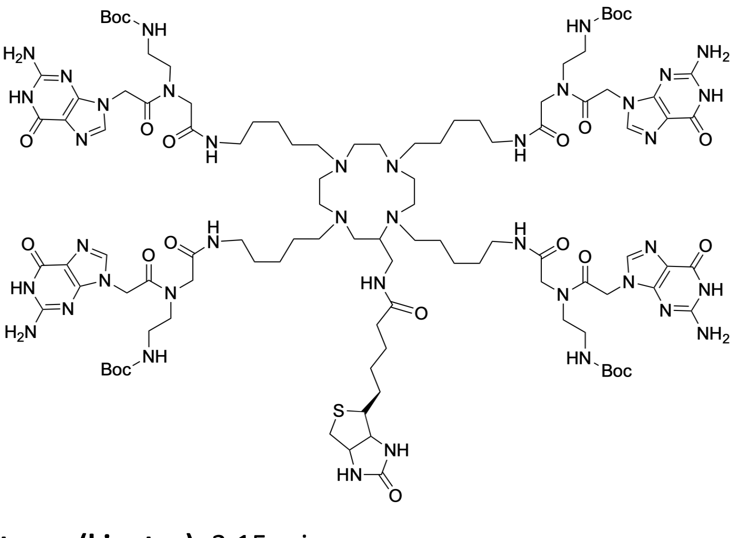

tr_{LCMS} (kinetex): 3.15 min.
MS (ESI$^+$): $[M+2H]^{2+}$ m/z = 1168.2 (calculated for $[C_{103}H_{165}N_{39}O_{22}S]^{2+}$: 1167.14288).
ESI HRMS: $[M+H+Na]^{2+}$m/z = 1178.63633 (calculated for $[C_{103}H_{166}N_{39}O_{22}SNa]^{2+}$: 1178.63519).

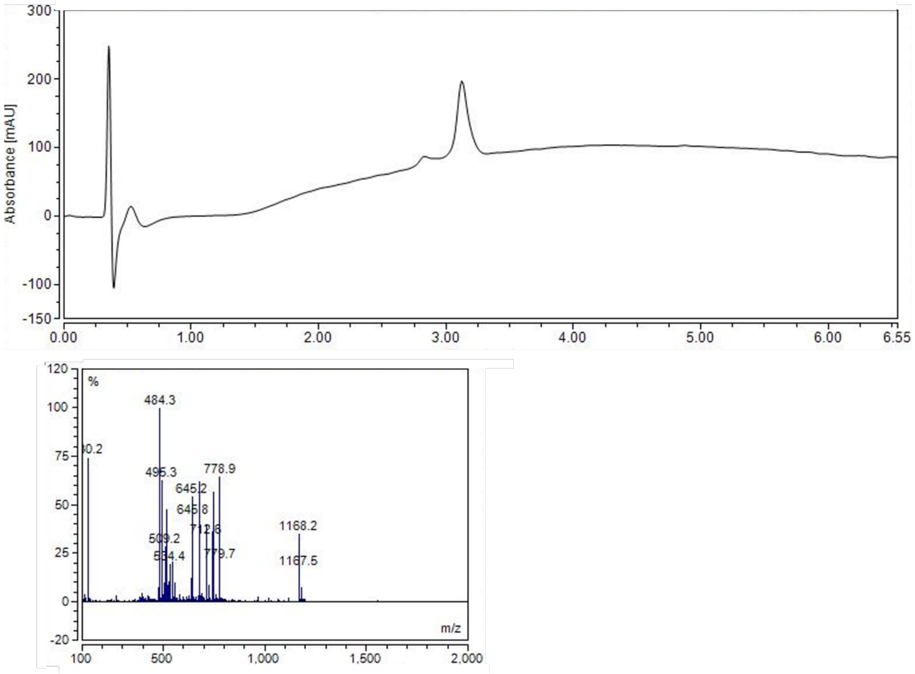

Figure S22: HPLC chromatogram and mass spectrum of protected BioCyTASQ

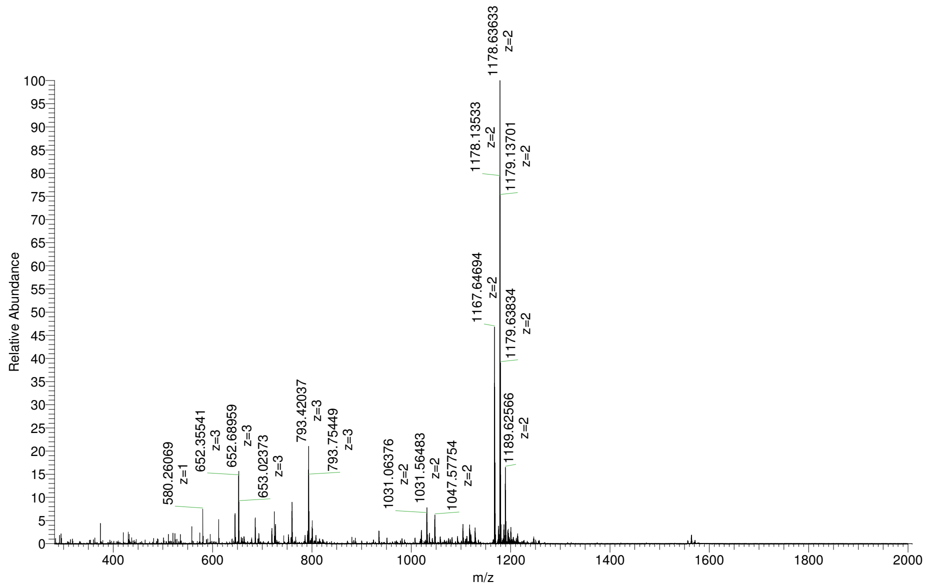

Figure S23: HRMS of protected BioCyTASQ

Protected MultiTASQ

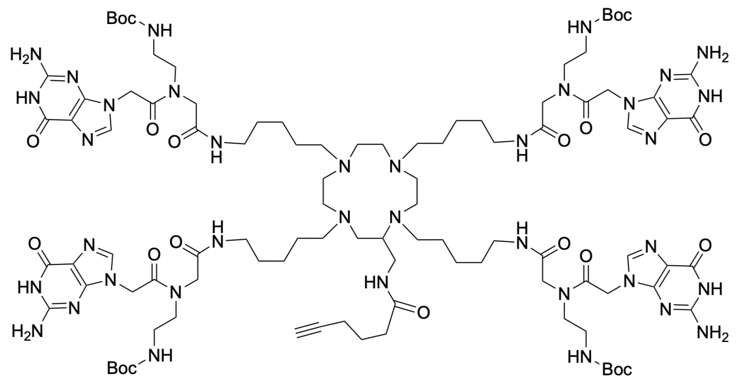

tr$_{LCMS}$ (kinetex): 3.20 min.
MS (ESI$^+$): [M+2H]$^{2+}$ m/z = 1102.3
ESI-HRMS: [M+2H]$^{2+}$ m/z = 1101.62541 (calculated for $[C_{99}H_{159}N_{37}O_{21}]^{2+}$: 1101.7995)

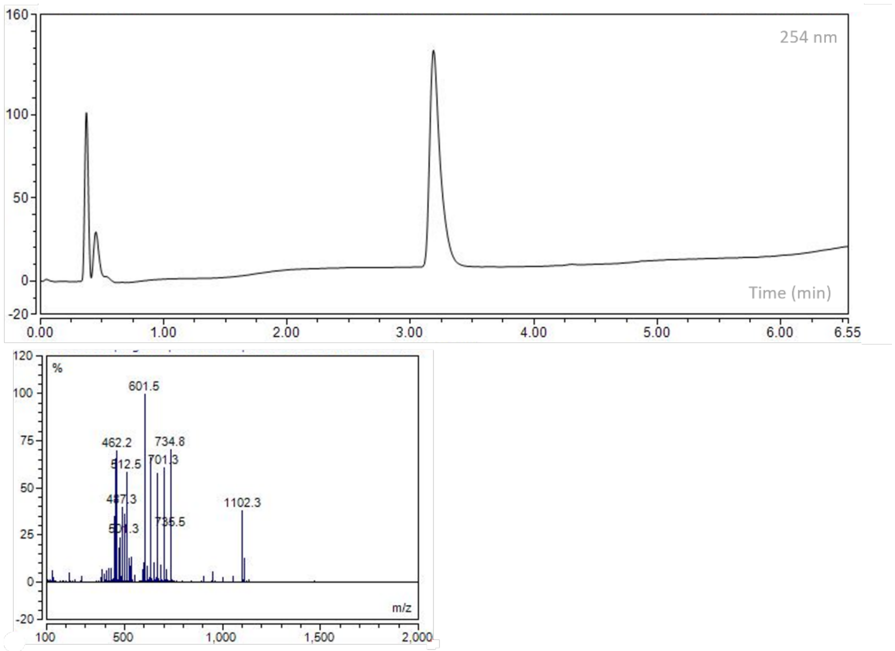

Figure S24: HPLC chromatogram and mass spectrum of protected MultiTASQ

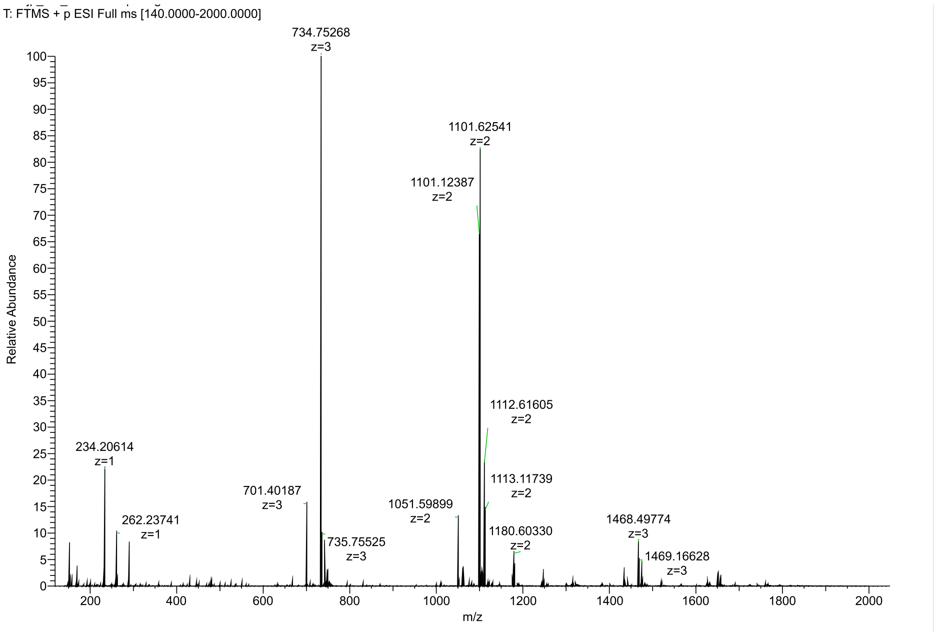

Figure S25: HRMS of protected MultiTASQ

Protected ^{az}MultiTASQ :

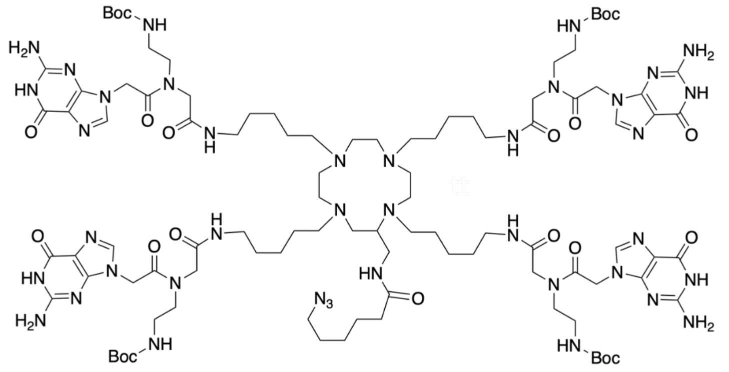

tr$_{LCMS}$ (kinetex): 3.22 min.
MS (ESI$^+$): [M+2H]$^{2+}$ m/z = 1124.7
ESI-HRMS: [M+2H]$^{2+}$ m/z = 1124.14250 (calculated for $[C_{99}H_{162}N_{40}O_{21}]^{2+}$: 1124.14304)

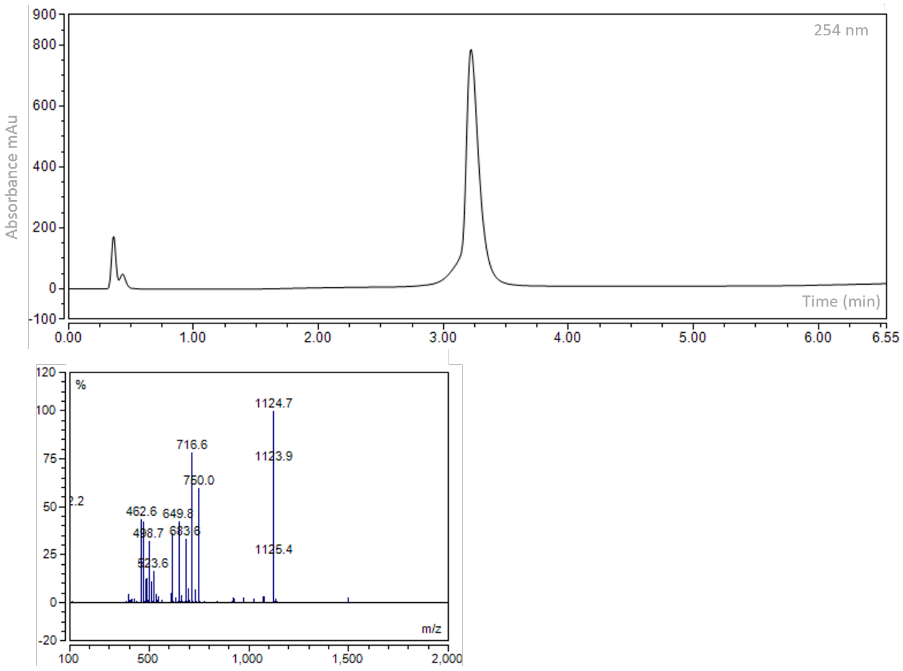

Figure S26: HPLC chromatogram and mass spectrum of protected ^{az}MultiTASQ

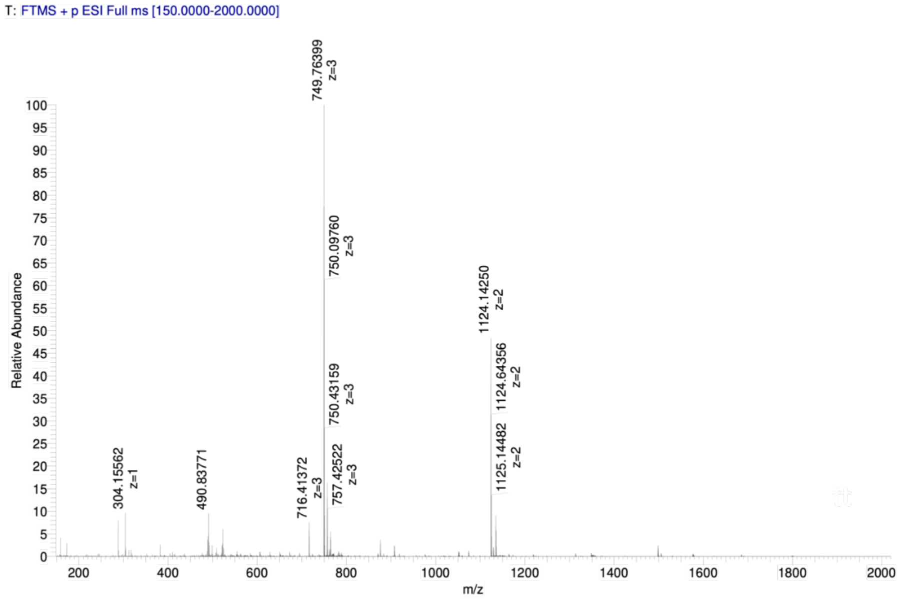

Figure S27: HRMS of protected ^{az}MultiTASQ

Protected photoMultiTASQ :

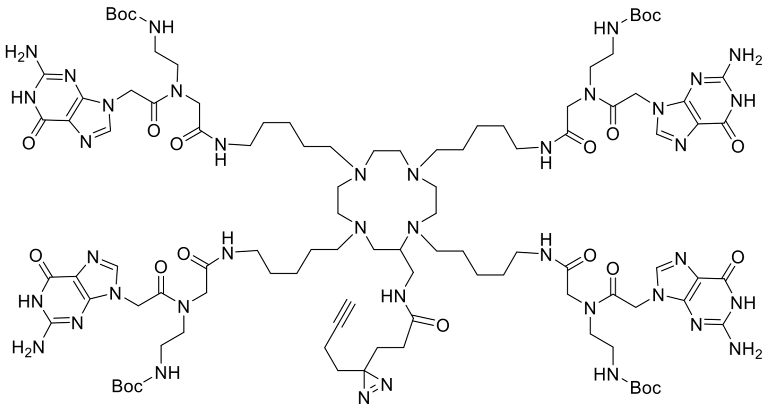

tr$_{LCMS}$ (kinetex): 3.20 min.
MS (ESI$^+$): [M+2H]$^{2+}$ m/z = 1128.8
ESI-HRMS: [M+2H]$^{2+}$ *m/z* = 1128.13458. (calculated for [$C_{101}H_{161}N_{39}O_{21}$]$^{2+}$: 1128.136775)

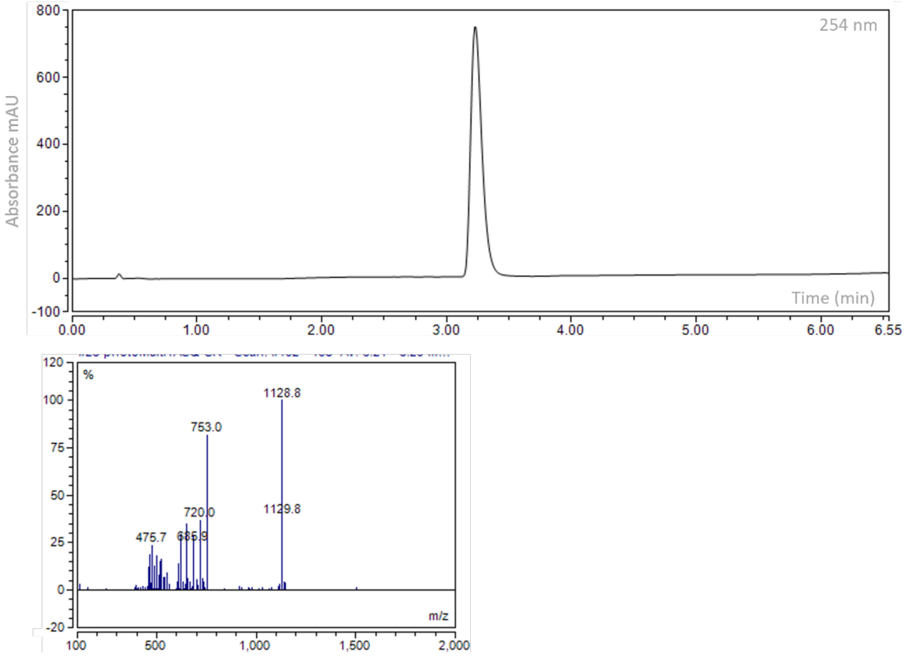

Figure S28: HPLC chromatogram and mass spectrum of protected photoMultiTASQ

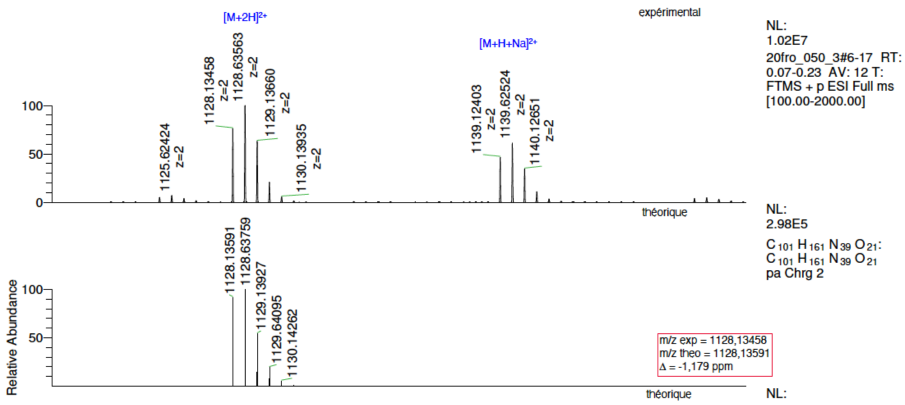

Figure S29: HRMS of protected photoMultiTASQ
